# Transcriptome-Guided Identification of Pectin Methyl-Esterase-Related Enzymes and Novel Molecular Processes Effectuating the Hard-to-Cook Defect in Common Bean (*Phaseolus vulgaris* L.)

**DOI:** 10.3390/foods11121692

**Published:** 2022-06-09

**Authors:** Mary Esther Muyoka Toili, Ramon de Koning, Raphaël Kiekens, Nelson Ndumba, Samuel Wahome, Sylvester Anami, Stephen Mwangi Githiri, Geert Angenon

**Affiliations:** 1 Laboratory of Plant Genetics, Faculty of Sciences and Bioengineering Sciences, Vrije Universiteit Brussel, 1050 Brussels, Belgium; Mary.Esther.Muyoka.Toili@vub.be (M.E.M.T.); Ramon.De.Koning@vub.be (R.d.K.); Raphael.Kiekens@vub.be (R.K.); 2 Department of Horticulture and Food Security, School of Agriculture and Environmental Sciences, College of Agriculture and Natural Resources, Jomo Kenyatta University of Agriculture and Technology, Nairobi P.O. Box 62000-00200, Kenya; wahomewanjohi@ymail.com (S.W.); githirim@agr.jkuat.ac.ke (S.M.G.); 3 Department of Biochemistry, School of Biomedical Sciences, College of Health Sciences, Jomo Kenyatta University of Agriculture and Technology, Nairobi P.O. Box 62000-00200, Kenya; ndumbanelson@gmail.com; 4 Institute for Biotechnology Research, Jomo Kenyatta University of Agriculture and Technology, Nairobi P.O. Box 62000-00200, Kenya; Sanami@jkuat.ac.ke

**Keywords:** pectin methyl-esterase (PME), pectin methyl-esterase inhibitor (PMEI), *Phaseolus vulgaris*, hard-to-cook defect, RNA sequencing

## Abstract

The hard-to-cook defect in common beans is dictated by the ability to achieve cell separation during cooking. Hydrolysis of pectin methyl-esters by the pectin methyl-esterase (PME) enzyme influences cell separation. However, the contributions of the PME enzyme and the cell wall to the hard-to-cook defect have not been studied using molecular tools. We compared relevant molecular processes in fast- and slow-cooking bean varieties to understand the mechanisms underpinning the hard-to-cook defect. A PME spectrophotometric assay showed minor differences in enzyme activity between varieties. Meanwhile, a PME *HMMER* search in the *P. vulgaris* genome unveiled 113 genes encoding PMEs and PME inhibitors (PMEIs). Through RNA sequencing, we compared the gene expression of the PME-related genes in both varieties during seed development. A PME (*Phvul010g080300*) and PMEI gene (*Phvul005g007600*) showed the highest expression in the fast- and slow-cooking beans, respectively. We further identified 2132 differentially expressed genes (DEGs). Genes encoding cell-wall-related enzymes, mainly glycosylphosphatidylinositol mannosyltransferase, xyloglucan O-acetyltransferase, pectinesterase, and callose synthase, ranked among the top DEGs, indicating novel relations to the hard-to-cook defect. Gene ontology mapping revealed hydrolase activity and protein phosphorylation as functional categories with the most abundant upregulated DEGs in the slow-cooking bean. Additionally, the cell periphery contained 8% of the DEGs upregulated in the slow-cooking bean. This study provides new insights into the role of pectin methyl-esterase-related genes and novel cell wall processes in the occurrence of the hard-to-cook defect.

## 1. Introduction

The common bean (*Phaseolus vulgaris* L.) is an important pulse crop that could promote the global agenda to adopt healthy diets containing plant-based proteins [1]. Bean seeds contain up to 20–25% proteins, and thus have a great potential to provide a rich source of protein [2]. Beans also contain other components critical for a healthy diet such as slow-release carbohydrates, dietary fiber, a multitude of B-vitamins (such as folate), and several minerals such as iron, potassium, phosphorus, and magnesium [2]. When combined with grain cereals such as maize, they form a wholesome and inexpensive diet with a potential synergistic effect that could provide significant health benefits [3]. It is expected that the demand for common beans will increase over the years as a source of dietary protein for millions of people who cannot consume animal protein due to affordability, health implications, or preference [4]. Economically, high global consumption of up to 18,825 kt was reported in 2019 [5,6].

The potential of beans as an essential food to promote food security can be felt particularly in the developing countries that heavily rely on bean nutrition [2]. In Kenya, common bean consumption is estimated to be 14 kg per year per capita, and reported to be as high as 66 kg per year in the western regions of the country [2,7]. Preferences towards the types of beans to grow and consume also exist here. Rosecoco beans are preferred for consumption, generally prepared as a stew to be taken along with rice or mixed with maize and boiled to make the Githeri dish, which is popular in rural households and the low-income urban population [8]. Cultivation of Rosecoco beans requires fertile soils and high rainfall, conditions that are absent in a large section of the country, since 89% of Kenya is made up of arid and semiarid lands (ASALs) [9]. The commercial production of Rosecoco is also hampered by its susceptibility to bean rust and angular leaf spot [10]. Since the majority of bean growers are small-scale farmers with limited financial resources for pesticides and fertilizer purchases, this has led to the adoption of the more adaptable bean variety Pinto, which grows well in less-fertile soils with low amounts of rainfall, and is particularly tolerant to bean mosaic virus [10].

While farmers in Kenya have readily accepted growing the Pinto variety, consumers have a difficult choice because Pinto beans are prone to developing the hard-to-cook (HTC) defect, which imposes a prolonged cooking period to achieve a sufficiently palatable bean meal [11,12]. Conventionally, harvested beans are stored for prolonged periods due to their excellent shelf life and to ensure availability during the off-season. Consequently, the beans are dried to achieve a moisture content that is biologically and chemically safe for long-term storage [13,14]. During cooking, the time to achieve cell separation and softening is considerably longer in beans with the HTC defect than in regular beans [15,16]. Apart from the increased energy demand required in preparing HTC beans, the HTC defect also negatively impacts beans’ cooking and processing quality, resulting in grainy beans with an unsatisfactory texture on the palate. Loss of vital nutrients has also been associated with prolonged cooking [17]. Moreover, the Food and Agricultural Organization (FAO) reported that the consumption of beans is currently hindered because the cooking time is much more than that of other foods such as vegetables [18].

The HTC defect is believed to occur due to a combination of factors, including the genetic composition of the beans, the growth environment, and the storage conditions of harvested beans [16]. However, considerable variation exists among different varieties concerning the cooking time, pointing towards a substantial genetic influence [16,19,20,21]. Moreover, despite the growing and storage conditions, bean types with an inherent HTC defect will always take considerably longer to cook than the ‘easy-to-cook’ (ETC) varieties [19,20,21]. 

The mechanisms underpinning the development of the HTC defect remain largely unknown. Several theories have been proposed, including the widely accepted theory in which the hardening of pectin in the cell wall results from pectin binding to divalent cations [12,22]. Unfavourable postharvest storage conditions of beans in high temperature of above 25 °C and relative humidity greater than 65% causes an enzyme-catalyzed reaction in which the pectin methyl-esterase (PME) enzyme catalyzes the hydrolysis of highly methyl-esterified pectin within the cell wall, resulting in de-methyl-esterified pectin, which readily binds to divalent cations in a blockwise pattern, causing hardening of the pectin molecules. The activity of PME is primarily regulated by pectin methyl-esterase inhibitors (PMEIs). However, both enzymes belong to large multigene families that are yet to be characterized in *P. vulgaris,* and require robust molecular tools to identify the contribution of specific members of the gene families to the HTC defect. 

Previous studies aiming to understand the HTC defect relied on the physical, chemical, and ultrastructural comparison of fast- and slow-cooking beans, in which the changes in the cell wall have been extensively studied [19,23,24,25,26]. Breeding studies have utilized quantitative trait loci (QTL) mapping and genome-wide association studies (GWAS) to identify chromosomal regions on *P. vulgaris* associated with cooking time. These studies have determined cooking time as an oligogenic and highly heritable trait, with several QTLs associated with cooking time mapped to eight of eleven chromosomes in the common bean genome [20,21,27]. Accordingly, a gene-expression database against which breeders compare the expression of essential genes would add value to the study of the HTC phenomenon for *P. vulgaris*. Currently, a transcriptome analysis in which the whole seed-development process was investigated, ranging from the early seed development to the mature seed stage of fast- and slow-cooking bean varieties, is absent. Furthermore, important questions as to whether there are any differentially expressed genes (DEGs) related to the cooking time remain largely unanswered. 

To understand the contribution of PME activity in the hard-to-cook defect, we performed a biochemical analysis with PME extracted from seeds of contrasting phenotypes of *P. vulgaris*. However, no significant difference was observed between the fast- and slow-cooking varieties, necessitating the application of more precise molecular tools. We therefore utilized RNA sequencing, which allows for the simultaneous study of the expression of thousands of genes and related isoforms (such as the PME gene family), which may be impossible in systematic gene-expression study using quantitative RT-PCR (qRT-PCR). The resulting gene-expression analyses across the fast- and slow-cooking biological conditions allowed for specific identification of the gene expression of several PME homologues and DEGs that act as potential molecular biomarkers of the HTC defect. Relevant DEGs identified in this study between fast- and slow-cooking beans related to cooking time will provide a valuable contribution to the molecular knowledge of the HTC defect. Finally, researchers can use the gene-expression data collected during progressing stages of seed development (from early to late maturity) in future experiments studying seed development in *P. vulgaris* and other legumes. 

## 2. Materials and Methods

### 2.1. Plant Material

This study used two Andean commercial bean genotypes previously characterized for cooking time [24]. Rosecoco (GLP 2) is a fast-cooking bean variety and one of Kenyans’ most preferred bean varieties for consumption [24,28]. On the other hand, Pinto (GLP X92) is a slow-cooking bean variety, but is adaptable to various agroecological zones and is drought tolerant [8,10,24]. The seeds were a kind donation of varieties that were already characterized for cooking time [24] that were initially obtained from the National Genebank of Kenya (NGK) of the Kenya Agricultural and Livestock Research Organization (KALRO-Kenya). Seeds were grown to maturity in a glasshouse at Vrije Universiteit Brussel (VUB), Belgium. Flowers were tagged on the date when they fully opened, and seeds were harvested at different stages of seed development, as counted according to the number of days after flowering (DAF). The sampling dates were 15, 20, 25, 30, 35, and 40 DAF. Harvested seeds were placed in tightly capped centrifuge tubes, immediately frozen in liquid nitrogen, and stored at −80 °C.

### 2.2. Pectin Methyl-Esterase-Coupled Enzymatic Assay

The PME enzyme was first isolated from sampled seeds at 20, 30, and 40 DAF in the form of soluble protein extract. An extraction buffer containing 100 mM Tri-HCl (pH 7.5), 500 mM NaCl, and 1x protease inhibitor cocktail (Sigma-Aldrich, Saint Louis, MO, USA, catalogue number P-9599) was added to 100 mg of frozen ground seed powder in a double amount (*w*/*v*) and vortexed for 10 s. Extracts were rotated at 4 °C for 30 min and centrifuged at 11,500× *g* at 4 °C for 20 min, and the supernatant (which contained soluble proteins in the extract, including the PME enzyme) was collected and immediately used in the PME assay [29,30]. A coupled enzyme assay was performed according to a procedure by Grisc-Rausch and Rausch (2004) with modifications. The methanol released from methyl-esterified pectins due to PME enzymatic activity was oxidized to formaldehyde using alcohol oxidase [31]. The formaldehyde was then used as an electron donor with formaldehyde dehydrogenase to reduce NAD+ to NADH. The formation of NADH was measured spectrophotometrically to estimate the PME activity in the protein extract. To initiate the reaction, 10 µL of the protein extract (containing the PME enzyme) was added to 180 µL master mix composed of 20 µL of 5% (*w*/*v*) pectin (Sigma Aldrich, Saint Louis, MO, USA, catalogue number P-9135) in H_2_O, 2 µL (1.0 U) alcohol oxidase from *Pischia pastoris*, (Sigma Aldrich, Saint Louis, MO, USA, catalogue number A-2404), 2 µL (0.35 U) formaldehyde dehydrogenase from *Pseudomonas* sp. (Sigma Aldrich, Saint Louis, MO, USA, catalogue number F1879), and 156 µL of 0.4 mM β-nicotinamide adenine dinucleotide—NAD+ (Sigma-Aldrich, Saint Louis, MO, USA, catalogue number N-8410) in 50 mM phosphate buffer (pH 7.5) in microplate wells. The positive control well contained 10 µL (7.8 mU) pectinase from *Aspergillus aculeatus* (Sigma Aldrich, Saint Louis, MO, USA, catalogue number E-6287) and protein extract buffer added to the master mix, while the background controls were composed of 10 µL of protein extract buffer added to the master mix. For the negative control, only 190 µL of the protein extract buffer was added to the well. Changes in NADH absorption were recorded at a wavelength of 340 nm for 10 min at room temperature (21–25 °C). The change in absorption per unit time (represented by the slope of the curve) was used to calculate the increase in concentration of NADH using the Beer–Lambert law, for which the extinction coefficient for NADH was ε340 = 6220 M^−1^cm^−1^, and 1 nkat PME activity was equivalent to 1 nmol NADH formed per second.

### 2.3. Identification of PME-Related Genes in P. vulgaris

A validated PME gene from *Solanum lycopersicum* (NP_001234151.1) and a PMEI gene from *Actinidia deliciosa* (AB091088) were used as queries in an NCBI protein BLAST to generate a list of PME-related genes of *P. vulgaris* [32]. Sequences that were not PME-related with regard to their functional annotation were discarded from the list. The remaining sequences were used for a multiple sequence alignment using MUSCLE in the MEGA software (v10.0.5) [33,34]. This alignment was used to build a HMMER motif using the *Hmmbuild* software (*HMMER* V3.1b2). The resulting plant-specific PME/PMEI-HMMER motif was subsequently used as input for the *Hmmsearch* software (*HMMER* V3.1b2) to search for PME/PMEI-related sequences within *P. vulgaris*. A total of 119 significant genes were recovered in the *Hmmsearch* (bit score > 250); these were manually filtered to 113 PME-related genes that were used as queries in the Phytozome PhytoMine tool to confirm their functions [35]. A heat map was constructed using TBtools software to visualize the gene expression of all the PME-related genes [36]. The genes’ molecular properties, including the theoretical isoelectric point (pI) and molecular weight, were computed using ExPASy tool [37]. The 113 genes were further filtered to keep only the genes with an average expression of >1 RPKM across any sampled development stage. The FASTA sequences of the 18 recovered genes were used to construct a phylogenetic tree using MEGA software (v10.0.5) after a multiple sequence alignment using MUSCLE [33,34]. The tree was composed of *P. vulgaris* PME-related genes and additional validated or provisional PME-related genes from *Arabidopsis thaliana* (NP_566038.1, NP_188048.1, NP_175786.1, NP_180701.1, and NP_188348.1), *Citrus sinensis* (NP_001275859.1, NP_001275775.1), *S. lycopersicum* (NP_001234151.1, NP_001303860.1) and *A. deliciosa* (AB091088, AB091089.1). The neighbor-joining (NJ) algorithm was used with the standard settings, and the bootstrap value was set to 1000. The TBtools software was used to construct a heat map of the 18 genes using RPKM gene-expression values [36]. The exon-intron gene model structures were constructed using the Gene Structure Display Server (GSDS v2.0) software and manually appended onto the respective phylogenetic trees [36,38].

### 2.4. RNA Isolation, Library Preparation, and Sequencing

RNA was isolated from four seed replicates sampled at 15, 20, 30, and 35 DAF using the RNeasy PowerPlant Kit (Qiagen, Hilden, Germany, catalogue number 13500-50). RNA concentrations and purity were estimated from the A260/280 absorbance ratio with a NanoDrop Spectrophotometer ND-1000 (Thermo Fisher Scientific, Wilmington, NC, USA). RNA integrity was checked on a 1% agarose gel using bleach gel electrophoresis [39]. RNA samples were submitted to the Genomics Core Leuven, where library preparation was performed using Lexogen’s QuantSeq kit (3‘mRNA-Seq Library Prep Kit FWD, Lexogen, Vienna, Austria). The QuantSeq protocol generates only one fragment per transcript from polyadenylated RNA, resulting in highly accurate gene-expression values. The sequences obtained were close to the 3′ end of the transcripts. The sequencing was then performed at the same facility using Illumina’s HiSeq4000 to generate single-end (SE) reads for all 32 prepared libraries (4 development stages × 4 biological replicates × 2 *P. vulgaris* varieties). However, only read counts of 24 RNA libraries (4 development stages × 3 biological replicates × 2 *P. vulgaris* varieties) that passed the initial quality control were utilized in this study. 

### 2.5. RNA Sequencing Pipeline 

The pipeline workflow used to obtain count files was based on the QuantSeq Data Analysis Pipeline of BlueBee^®^ Genomics, which is adapted specifically for the analysis of QuantSeq 3′ mRNA-Seq data [40]. Raw reads were received as FASTQ files and subjected to quality control using FastQC (v0.11.8) [41]. Adapter contamination, polyA readthrough, and low-quality tails were removed using the BBDuk program from the BBmap suite (v38.50b). Default settings were maintained as follows: k = 13; ktrim = r; useshortkmers = t; mink = 5; qtrim = r; trimq = 10; and minlength = 20. A second quality control was performed on the trimmed reads using FastQC (v0.11.8) [41]. Clean reads were mapped to the common bean reference genome for *P. vulgaris* available on Phytozome (Assembly v2.0 and Annotation v2.1) using STAR (v2.6.0c), after which the Qualimap software (v2.2.1) was used to perform quality control of the mapped reads [42,43,44]. Final read files with the lowest percentage of uniquely mapped reads for each biological replicate set were filtered out from further analysis, resulting in three biological replicates for each sampled seed-development stage. Count files were generated using HTSeq-count (v0.9.1), and the data were normalized using different methods depending on the use of the data [45]. 

### 2.6. Identification of Differentially Expressed Genes

Raw counts (without normalization) were loaded into OmicsBox software (v1.4.11) from Biobam for downstream analyses [46]. A simple design for differential expression analysis (DEA) was conducted using EdgeR (v3.28.0) [47]. Genes with zero counts in all samples were eliminated in the analysis and weighted trimmed mean of M-values (TMM) normalization was applied. TMM is useful for intersample comparisons, but does not correct the observed read counts for the gene length, which is theoretically irrelevant for inter-sample comparisons [48]. For this normalization, count data were trimmed: 5% for the A values, and log-ratio 0.3 for the M values to a reference array (the library whose upper quartile was closest to the mean upper quartile), after which scaling factors for each sample were generated using the *calcNormFactors* function in EdgeR. Scaling factors were then used to adjust the total mapped reads count from each sample. A multifactorial design for differential expression analysis was used to identify DEGs at different development stages using EdgeR [47]. A generalized linear model (GLM) likelihood ratio statistical test was used in the differential expression analysis. Statistics related to reads per sample were generated in a bar chart, and sample association was displayed using multidimensional scaling plots in unsupervised clustering. 

For all analyses (unless otherwise stated), the DEGs were those genes with a log2 fold change of <−1 and >1, and significant at an FDR of <0.05. These upregulated and downregulated genes were used to construct a volcano plot and an MA plot (log ratio, M against log mean values, A) in OmicsBox [46]. All DEGs between Pinto and Rosecoco samples at 15 DAF, 20 DAF, 30 DAF and 35 DAF were listed separately and used to construct Venn diagrams showing shared DEGs between stages. An interactive cluster heat map was constructed using gene expression count data normalized to counts per million (CPM) within OmicsBox [46]. The counts per gene were normalized to CPM by dividing them by the total number of mapped reads per sample and multiplying by 1 × 10^6^ [48]. The CPM normalized data were then transformed with log2 using an offset of 1. CPM normalization helps to compare replicates of the same sample group, giving essential insights into the gene-expression patterns between replicates in a heat map. The heat map was constructed using the TBTools software with rows and columns clustered using the average cluster method and Euclidean distance [36]. Normalized scaling was applied to rows.

### 2.7. Functional Annotation of Differentially Expressed Genes

FASTA nucleotide sequences of all DEGs were retrieved using the Phytozome (v12.1.6) data-mining tool Phytomine by using the *P. vulgaris* gene identifiers (IDs) as queries [35,42]. These sequences were used in a BLASTp analysis in OmicsBox by utilizing the software’s Cloud BLAST on the nonredundant protein sequences (nr v5) database [46]. The taxonomy filter for Phaseolus (3883) was selected, and the default parameters were maintained for the rest of the filters. To retrieve the protein domains and/or motifs within the sequences, InterProScan (v5.50-84.0) was run against several databases, including CDD, HAMAP, HMMPanther, HMMPfam, Gene3D, SFLD, Superfamily, and MobiDBLite (among others) [49]. A gene ontology (GO) mapping was performed to retrieve GO terms associated with the BLAST hits, and annotation was then performed against GO Gene Annotation Files and UniProt ID-Mapping to assign functional labels to the sequences [50]. Default parameters were maintained for mapping and annotation. GO multilevel pie charts combining similar sequences were then generated from the resulting GO functions.

### 2.8. Enrichment Analysis of Differentially Expressed Genes

Fisher’s exact test was used to identify over-represented and under-represented sequences in the DEGs. Upregulated or downregulated sequences were compared to a reference annotation for *P. vulgaris* (*P. vulgaris*_442_v2.1) available on Phytozome using the FatiGO package for statistical assessment of annotation differences [42,51]. The *p*-values were corrected for multiple testing using the false discovery rate (FDR) according to the Benjamini–Hochberg procedure [52]. 

### 2.9. Validation of RNA Sequencing Data by qRT-PCR

Three biological replicates of cDNA samples from Pinto and Rosecoco varieties at 15, 20, 30, and 35 DAF were used for qRT-PCR on the CFX96 Touch^TM^ Real-Time PCR Detection System (Bio-Rad, Singapore). The GoTaq^®^ qPCR Master Mix (Promega, Madison, WI, USA, catalogue number A6001) was used for the fluorescence assay, which combined a proprietary dsDNA-binding dye (BRYT Green^®^ Dye), a low level of carboxy-X-rhodamine (CXR) reference dye, GoTaq^®^ Hot Start Polymerase, MgCl_2_, dNTPs, and an optimized reaction buffer. The following amplification settings were used: initial heating at 95 °C for 3 min followed by 40 cycles of each 15 s at 95 °C and 1 min at 60 °C. The fluorescence was measured after each cycle. The comparative ΔCT method was used to normalize the cDNAs threshold cycle (Ct) values observed with qRT-PCR using *B-tubulin* as a reference gene [53]. Differential expression of genes of interest between the Pinto and Rosecoco varieties was quantified using the 2^−ΔΔCt^ method [54]. Logarithmic normalization (log base 2) of the fold-change values was performed to make comparisons with log2 fold-change values obtained from the RNA sequencing data using DESeq2 (v1.30.1) in R Studio (v1.2.1335) [55,56,57]. Primers used were subjected to a standard curve analysis to validate the specificity and amplification efficiency of each primer pair; the sequences used are listed in Supplementary Table S1. 

### 2.10. Statistical Analysis

Means were analyzed using one-way analysis of variance (ANOVA), and post hoc comparisons of means were made using the Fisher’s least significance difference (LSD) test in R Studio (v1.2.1335), where applicable [56,57]. Differences were considered statistically significant at *p* < 0.05.

## 3. Results

### 3.1. Characterization of PME Activity in Fast- and Slow-Cooking Bean Varieties Using a Coupled Enzymatic Assay

**Figure 1:**
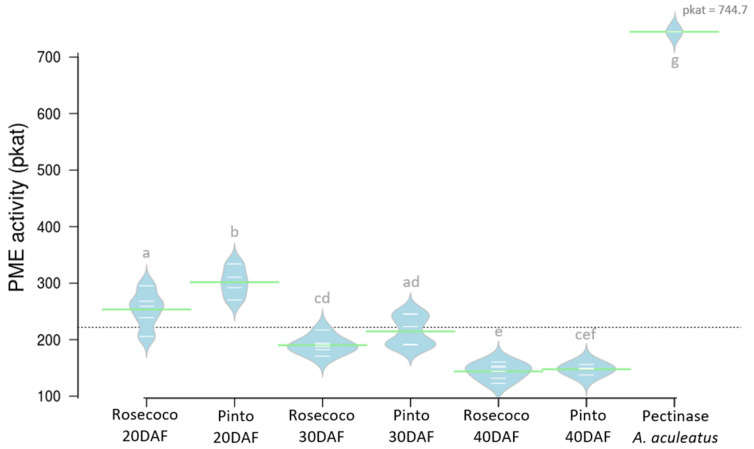
Bean plot showing the activity of the PME enzyme in developing seeds of fast-cooking (Rosecoco) and slow-cooking (Pinto) *P. vulgaris* varieties. Green lines indicate the sample means, white lines represent individual data points, and polygons represent the estimated density of the data. The dashed horizontal line shows the overall mean. Means with similar letters were not significantly different at *p* < 0.05.

A coupled spectrophotometric assay revealed differences in the utilization of commercially available pectin isolated from citrus peel by PME enzymes found in protein extracts of the fast- and slow-cooking beans (Figure 1). There was a very high activity of PME (pkat = 744.7) in the positive control experiment, which was composed of commercially available pectin under hydrolysis by pectinase from *A. aculeatus*. In the beans, we observed that the average PME activity decreased with an increase in seed age. When computed, the PME activity also declined with increased seed age in both bean varieties. However, there were no significant differences in the activity of PME between varieties except at 20 DAF, where PME activity was higher in Pinto bean (pkat = 301.7) compared to Rosecoco (pkat = 253.4). 

### 3.2. Characterization of PME-Related Genes in P. vulgaris Using Bioinformatic Tools

Using bioinformatic tools, we established that there existed several homologues of the PME genes, including PME inhibitors (PMEIs), that may all have contributed to the activity observed in Figure 1. A total of 113 genes with similar protein motifs to a validated PME gene from *S. lycopersicum* (NP_001234151.1) and a PMEI from *A. deliciosa* (AB091088) were recovered from a *HMMER* search in the *P. vulgaris* genome (*P. vulgaris*_442_v2.1). The functional description and domain search of these 113 genes showed they were all either PMEs or PMEIs. 

**Figure 2:**
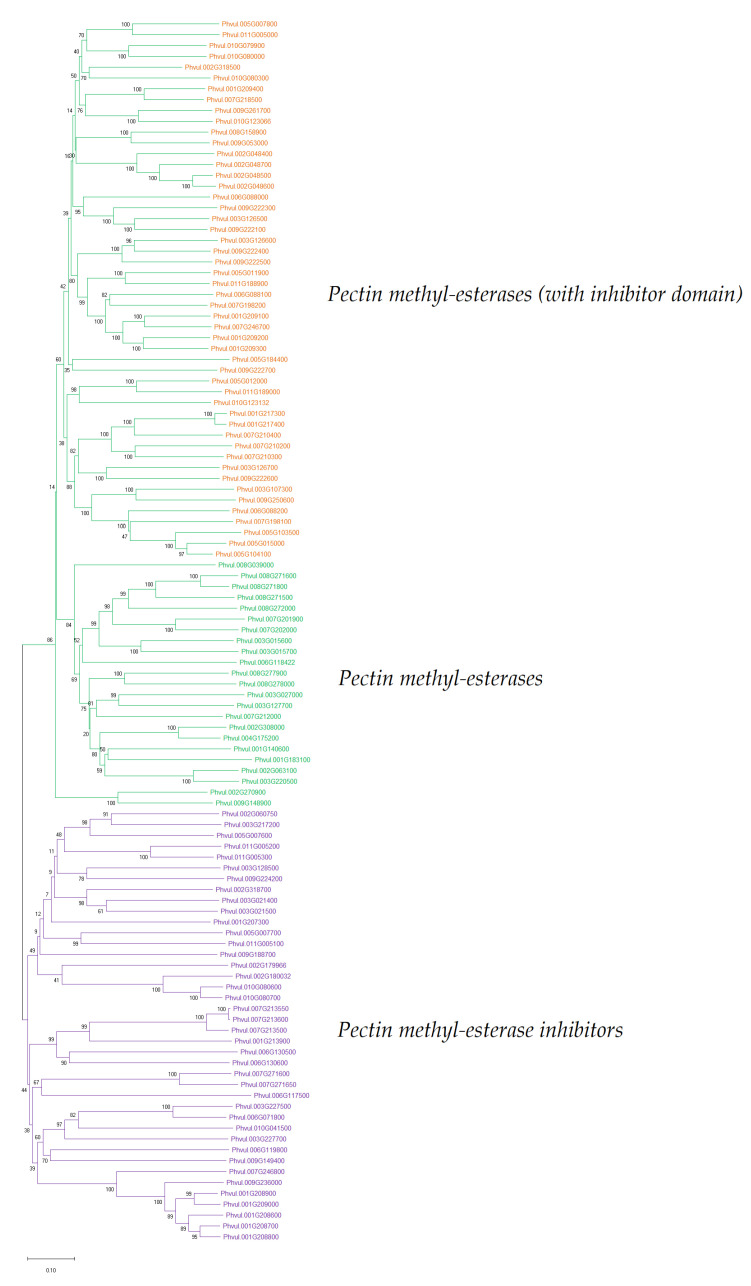
Phylogenetic tree showing the evolutionary relationship of a total of 113 genes encoding pectin methyl-esterases and pectin methyl-esterase inhibitors in *P. vulgaris*. The tree was inferred using the neighbor-joining method, and the evolutionary distances were computed using the p-distance method in MEGA software [34].

The phylogenetic tree of all *P. vulgaris* PME-related genes is shown in Figure 2. Here, the PME-related genes clustered into three subclasses composed of 47 pectin methyl-esterases with an inhibitor domain (orange cluster), 27 pectin methyl-esterases without an inhibitor domain (green cluster), and 39 pectin methyl-esterase inhibitors (violet cluster). In total, there were 74 PMEs and 39 PMEIs. The theoretical pI (isoelectric point) of the PME-related genes ranged from 4.21 to 11.12, while their molecular weights varied between 9133.4 and 69,117.0 kDa. The complete list of the genes, including their molecular properties such as pI and molecular weight, is shown in Supplementary Table S2.

### 3.3. Comparison of Gene Expression of PME and PMEIs in Fast- and Slow-Cooking Bean Varieties 

We performed an RNA sequencing experiment to identify the expression of the multiple PME-related genes in *P. vulgaris* in order to understand their individual contribution to the PME activity in the fast- and slow-cooking beans. Single-end read sequencing generated a total of 141,365,247 reads (Table 1) from a combination of 24 RNA libraries isolated from the slow-cooking Pinto variety and fast-cooking Rosecoco variety at 15, 20, 30, and 35 DAF, providing a vast number of reads for the subsequent mapping. A high number (mean of 80.82%) of these reads were uniquely mapped to the *P. vulgaris* reference genome (*P. vulgaris*_442_v2.1) [42]. This coverage increased the possibility of accurately identifying genes corresponding to these unique reads. Furthermore, most of the reads mapped to exon regions of the genome, allowing us to identify gene-coding sequences that were useful in identifying DEGs (Supplementary Table S3). The reads that mapped to gene-coding regions of the genome were subsequently scored to obtain 106,348,694 raw read counts, ranging from a minimum of 2,878,112 to a maximum of 7,374,463 per library (Table 1).

Quality-control statistics of sequence reads and mapped counts were obtained using FASTQC (v0.11.8) and Qualimap (v2.2.1) software analyses [41,44].

**Figure 3:**
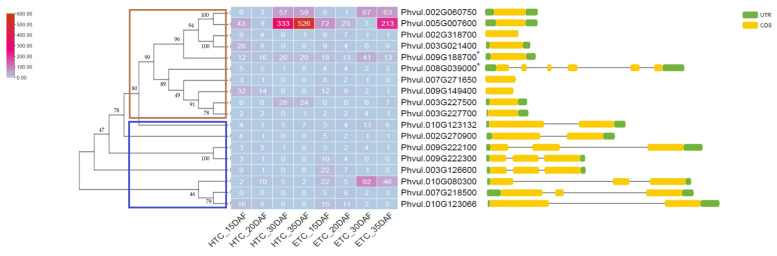
A hierarchical clustered heat map showing the RPKM expression and the gene structure of highly expressed genes encoding the pectin methyl-esterases (PMEs) and pectin methyl-esterase inhibitors (PMEIs) from fast-cooking (Rosecoco, ETC) and slow-cooking (Pinto, HTC) *P. vulgaris* varieties. Brown and indigo boxes indicate the PMEI and PME clusters, respectively. PME-encoding genes that clustered together with PMEIs are denoted by indigo asterisks. UTR: untranslated region; CDS: coding sequence.

The gene-expression values of the 113 previously identified PME-related genes were retrieved from the RNA sequencing analysis. These genes showed varying levels of expression of PMEs and PMEIs in the fast- and slow-cooking beans. Gene expression of the PME-related genes was generally low for genes in both bean varieties. Only 18 genes exhibited an average expression value of >1 RPKM in all seed-development stages of 15, 20, 30, and 35 DAF, which were sampled as shown in the heat map in Figure 3.

A hierarchical dendrogram appended to the heat map divided the 18 genes into two main clusters composed of PMEs and PMEIs, as clearly illustrated by the attached protein motifs retrieved using the NCBI CDD Batch Search tool [38,58]. However, two genes (*Phvul.009g188700* and *Phvul.008g039000*) encoding the PMEs clustered together with the PMEIs; these are indicated by an indigo asterisk on the heat map (Figure 3). *Phvul.005g007600* was the most expressed PMEI, with an almost 2-fold higher expression in the slow-cooking bean when compared to the fast-cooking bean at 35 DAF. While nearly no expression was observed for the same gene in the fast-cooking bean at 30 DAF, a high expression of 333 RPKM was observed in the slow-cooking bean at the same stage of seed development. Another interesting PMEI was *Phvul.003g227500,* which exhibited expression of 28 and 24 RPKM in the slow-cooking bean at 30 and 35 DAF, respectively, and almost no expression (0 and 7 RPKM) at the same seed-development stages in the fast-cooking bean. *Phvul.002g060750* is a PMEI that showed an almost similar expression in both bean varieties, with increased expression at 30 and 35 DAF.

On the other hand, the PME *Phvul.010g080300* was notably expressed highly in the fast-cooking bean, particularly at 30 and 35 DAF, and the PME *Phvul.009g188700* also showed a higher expression in the fast-cooking bean compared to the slow-cooking bean (RPKM of 41 at 30 DAF), while the expression between the two phenotypes was comparable in the rest of the seed-development stages. The PME *Phvul.009g222300* was more expressed in the fast-cooking bean at 15 DAF, while the expression of *Phvul.010g123066* was comparable in both phenotypes. Overall, the PMEs seemed to be expressed more in varying stages of seed development in the fast-cooking bean, while the expression of the PMEIs was observed to be higher in the slow-cooking bean (Supplementary Figure S1).

### 3.4. Exon–Intron Structure of Highly Expressed PME and PMEI Genes from P. vulgaris in Comparison to Other Plant Species

**Figure 4:**
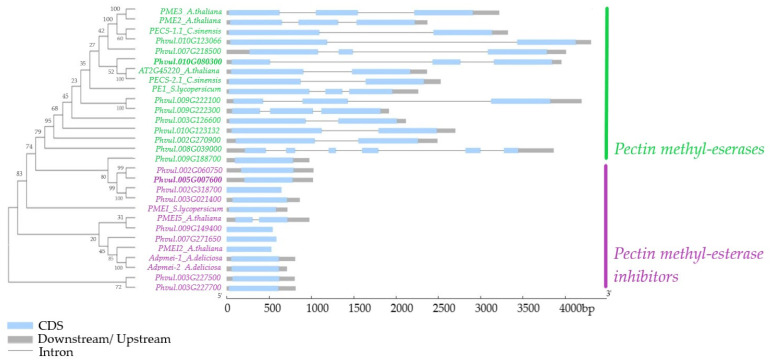
Phylogenetic tree showing the evolutionary relationship of highly expressed genes encoding pectin methyl-esterases (PMEs) and pectin methyl-esterase inhibitors (PMEIs) in *P. vulgaris* in comparison with those from other plant species (*A. thaliana*, *C. sinensis*, *S. lycopersicum,* and *A. deliciosa*).

In a phylogenetic analysis of the 18 highly expressed genes, a distinct classification of PMEs and PMEIs was observed. The first nine *P. vulgaris* genes clustered with the validated PME genes from *A. thaliana*, *C. sinensis,* and *S. lycopersicum* (Figure 4). The genes within this cluster were longer (over 1500 bp), with each having two to three exons except for one gene (*Phvul.008g039000*) with six exons. On the other hand, the *P. vulgaris* genes clustering with validated PMEI genes from *S. lycopersicum*, *A. thaliana*, and *A. deliciosa* showed a shorter length of less than 1500 bp, with each containing only one exon (Figure 4). The only gene with two exons in this cluster was *PMEI5* from *A. thaliana*. The exon–intron structure for the complete set of the 113 PME-related genes is shown in Supplementary Figure S2.

### 3.5. Gene-Expression Analysis of PME and PMEI Using Quantitative RT-PCR

**Figure 5:**
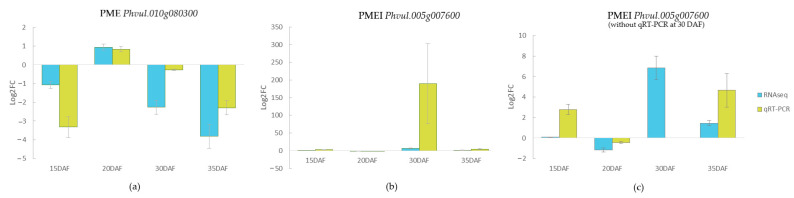
Bar plots showing a gene expression comparison in selected genes encoding (**a**) a pectin methyl-esterase (PME) *Phvul.010g080300* and (**b**) a pectin methyl-esterase inhibitor (PMEI) *Phvul. 005g007600* in an RNA sequencing and qRT-PCR experiment. The gene expression of PMEI *Phvul.005g007600* excluding the qRT-PCR gene expression visualization at 30 DAF is shown in (**c**). Gene expression is shown in log2 fold-change values (Log2FC) in the slow-cooking Pinto bean (treatment sample) when compared to the fast-cooking Rosecoco bean (control sample) with *β-tubulin* as the housekeeping gene [53]. Error bars represent the standard error within samples.

Two PME-related genes selected for confirmation of the RNA sequencing results showed similar gene-expression results using qRT-PCR (Figure 5). The genes, which included the highest expressed PME (*Phvul.010g080300*) and PMEI (*Phvul.005g07600*), showed similar patterns of upregulation or downregulation in the slow-cooking bean in a comparison between the RNA sequencing and qRT-PCR experiments, as illustrated in Figure 5a,b. The log2 fold-change (Log2FC) pattern for both experiments was similar for the PME gene *Phvul.010g080300,* in which we observed an upregulation of the PME gene at 20 DAF in the slow-cooking bean for both RNA sequencing and qRT-PCR, and a further downregulation at 15, 30, and 35 DAF. For the PMEI *Phvul.005g007600*, we observed a very high Log2FC of 189.53 at 30 DAF in the qRT-PCR experiment. An upregulation of 6.85 was also observed in the RNA sequencing experiment at the same seed-development stage. Overall for this gene, an upregulation of expression in the slow-cooking bean was observed at all sampled seed-development stages except for 20 DAF, for which a downregulation of −1.15 and −0.44 was observed in the RNA sequencing and qRT-PCR experiments, respectively (Figure 5b,c).

### 3.6. Differential Expression Analysis of Genes Expressed in Fast- and Slow-Cooking Bean Varieties

**Figure 6:**
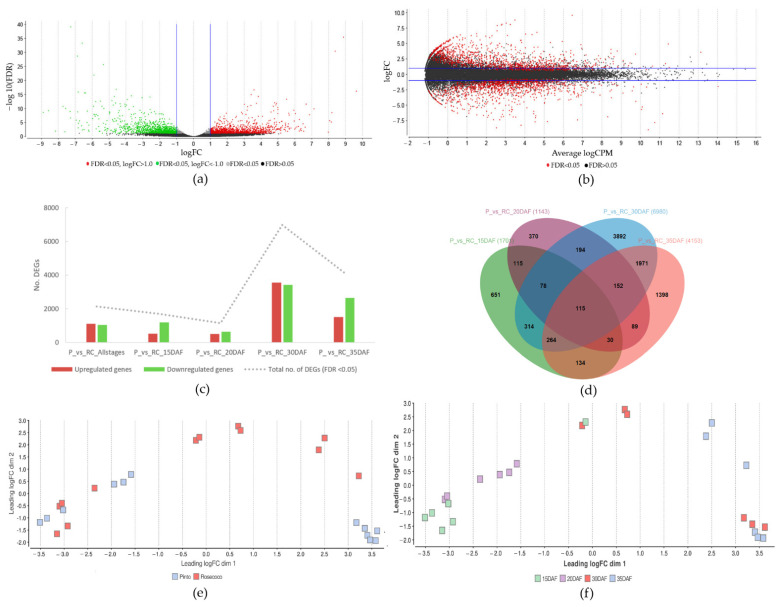
Results of the differential gene-expression analysis showing the distribution of samples and DEGs. (**a**) Volcano plot showing the log2 fold change in expression between slow-cooking beans (Pinto) and fast-cooking (Rosecoco) on the *x*-axis against the negative Log10 of FDR of the DEGs (*y*-axis). Significantly upregulated and downregulated genes are indicated with red and green data points, respectively. Black data points depict nonsignificant DEGs. (**b**) MA plot showing the log2 fold change in expression in fast- and slow-cooking beans (*y*-axis) against mean expression level (*x*-axis). Red data points depict DEGs that were significant at FDR < 0.05. Black data points had FDR values of >0.05. Blue lines depict the specified thresholds for the log2 fold change in (**a**,**b**). (**c**) Bar chart showing the number of DEGs in the samples, and (**d**) Venn diagram showing the number of shared DEGs between the samples. (**e**) Multidimensional scaling (MDS) plot showing the pairwise distribution of samples within the different phenotypes of slow-cooking and fast-cooking beans, and (**f**) within the different seed-development stages. Distances on the plot represent log2 fold changes between the samples.

A differential gene-expression analysis was conducted to identify DEGs between the fast- and slow-cooking varieties and different seed-development stages (Figure 6). In all differential gene-expression analyses, the slow-cooking bean (Pinto) represented the experimental condition, whereas the fast-cooking bean (Rosecoco) was the reference (or control) condition. As such, the upregulated genes were more expressed in the Pinto bean when compared to Rosecoco, whereas the downregulated genes were less expressed in Pinto, but more expressed in Rosecoco.

Using the differential expression analysis of the EdgeR (v3.28.0) software available within OmicsBox, an overall view of the differentially expressed genes between the varieties and seed-development stages was obtained, as illustrated in Figure 6a–c [46,47]. There were 2132 differentially expressed genes (DEGs) significant at a false discovery rate (FDR) <0.05 when a log2 fold change threshold of <−1 and >1 was applied (Figure 6a,b). This number represented 8.15% of all the genes analyzed, providing an ample number of genes to identify markers for the HTC defect. Out of these DEGs, 1097 were upregulated in the slow-cooking bean, while 1035 genes were downregulated (Figure 6c). 

A multifactorial differential expression analysis was further performed to provide insight into the DEGs observed between the varieties at distinct stages of seed development. Here, differential expression analysis was performed with different combinations of varieties and seed-development stages using the EdgeR software within OmicsBox [46,47]. Results of the comparison between Pinto and Rosecoco at 15 DAF, 20 DAF, 30 DAF, and 35 DAF, respectively, are shown in Figure 6c. At 15 DAF, the total DEGs was 1701, with 511 upregulated genes and 1190 downregulated genes. The lowest number of DEGs was observed at 20 DAF, for which 1143 genes were differentially expressed, signifying more similar gene-expression patterns for a considerable number of genes in the fast- and slow-cooking beans at this seed-development stage. Of the DEGs at 20 DAF, 503 were upregulated and 640 were downregulated. The highest number of DEGs (6980) was observed between Pinto and Rosecoco at 30 DAF, with 3553 upregulated and 3427 downregulated genes. At this stage (30 DAF), the upregulated genes in the slow-cooking variety (Pinto) were greater in number than the downregulated ones. This observation implied that the seed at 30 DAF represented the best stage to visualize and identify the highest number of DEGs between the fast- and slow-cooking beans. Lastly, at 35 DAF, the total DEGs was 4135, with 1501 upregulated and 2652 downregulated genes (Figure 6c). 

An overlap was observed between these DEGs in different seed-development stages, as illustrated in Figure 6d. A total of 115 genes were consistently up- or downregulated in the slow-cooking bean regardless of the development stage of the bean seed. This provided an interesting list of genes that were potentially useful to gather additional insight into the HTC phenomenon because they were not influenced by the development stage of the seed, and may thus be inherent in determining the phenotype of the bean.

During the differential gene-expression analysis, the intergroup and intragroup variability of the samples were also examined to confirm the consistency and thus reliability of the samples. Multidimensional scaling (MDS) plots showed an expected association between sample groups. Intergroup association was visible at the variety level, where all samples of Pinto and Rosecoco clustered in separate groups (Figure 6e). Intragroup clustering within sampled stages of development was also observed, except for one sample of Rosecoco at 15 DAF, which clustered with Rosecoco at 30 DAF (Figure 6f). This sample was excluded from further downstream data analysis. Samples of the Pinto variety also grouped together at 30 and 35 DAF. Clustering of samples indicated that a similar expression profile of genes was observed within these samples.

### 3.7. Functional Classification of Differentially Expressed Genes Identified between Fast- and Slow-Cooking Bean Varieties

**Figure 7:**
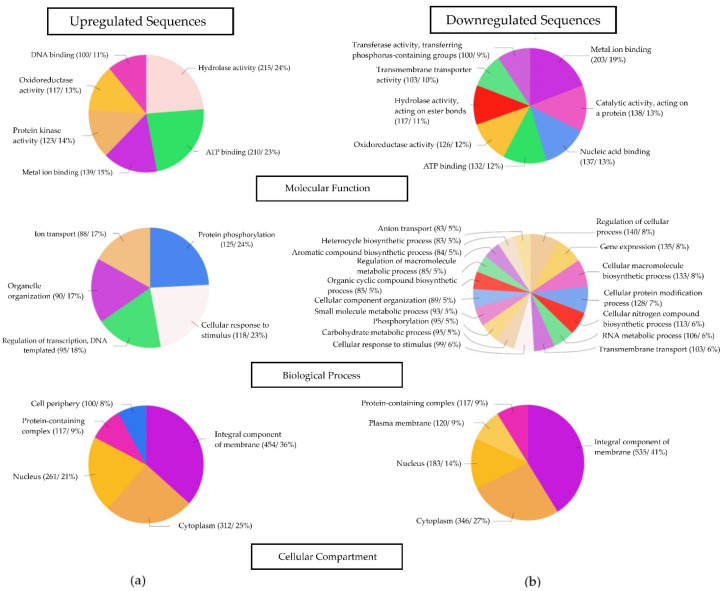
Gene ontology (GO) functional classification of DEGs associated with (**a**) upregulated genes and (**b**) downregulated genes of the slow-cooking bean (Pinto). GO categories were divided into molecular function, biological process, and cellular compartment. Only DEGs with a log2 fold change of <−1 and >1 and an FDR of <0.05 were included in the analysis.

While the study of individual gene expression patterns may allow for identifying specific genes of interest, the classification of groups of functionally related genes may provide a better insight into the overall contribution of a particular set of genes in the studied condition. Therefore, protein domains identified in the DEGs were used in a gene ontology (GO) mapping and functional annotation [50]. Different gene functions were linked to the DEGs, as shown in Figure 7. The GO terms associated with the molecular function, biological process, and cellular compartment of the up- and downregulated sequences are shown in Figure 7a,b, respectively.

Within the molecular function category, hydrolase activity in its broad form represented the GO term with the most abundant number of genes (24%) that were upregulated in the slow-cooking bean variety (Figure 7a). Equally, hydrolase activity (acting on ester bonds) was represented by 11% of the downregulated genes in the slow-cooking bean variety, indicating its importance to the fast-cooking bean (Figure 7b). Hydrolase activity (GO:0016787) is a broad GO term describing enzymes that catalyze the hydrolysis of various bonds. All enzymes within the Enzyme Commission (EC) class 3 belong to this group, including pectin methyl-esterases and phytases that have been linked to the HTC defect in the pectin-cation-phytate theory. Ideally, these enzymes should be highly expressed in the slow-cooking bean. However, this was not possible to conclude from this analysis due to the broad nature of the hydrolase enzyme group [59]. 

Metal ion binding is another broad GO term (GO:0046872) representing enzymes that interact selectively and noncovalently with any metal ion. It is a cation-binding GO term (GO:0043169) and is important in the theory regarding the development of the HTC defect [22]. Metal ion binding genes were present in both the up- and downregulated genes, with more genes (19%) present in the downregulated gene pool, suggesting importance in the fast-cooking bean (Figure 7b). 

There also existed groups of GO terms that were represented in both the up- and downregulated genes. For example, ATP binding, oxidoreductase activity, and DNA binding GO terms were present in both up- and downregulated genes, indicating the importance of these genes in both the fast- and slow-cooking bean varieties. However, the protein kinase functional group was only present in the upregulated genes, indicating potential usefulness in the slow-cooking bean, whereas transmembrane transporter activity and transferase activity (transferring phosphorus-containing groups) were only present in the downregulated genes, therefore suggesting a potential benefit to the fast-cooking bean.

While only 5 functional groups of the biological process GO category were present in the upregulated genes important to the slow-cooking bean variety, 17 functional groups were identified related to the down-regulated genes critical to the fast-cooking bean (Figure 7). The recovered GO terms in the biological process category varied when a comparison was made between the up- and downregulated genes, and the only shared GO term here was ‘cellular response to stimulus’. Shared GO terms indicated the importance of the genes within these groups in both the fast- and slow-cooking bean varieties. It was noteworthy, however, that many GO terms were derivations of previous GO terms on the GO hierarchy tree; i.e., they shared a common ancestor GO term. As such, they were inter-related, and may not offer distinct functional differences between up- and downregulated genes. 

Lastly, the cellular compartment GO category presented the least number of GO terms associated with the DEGs (Figure 7). Most of the identified GO terms were present in both the up- and downregulated genes except for the cell periphery GO term, which was present in the upregulated genes of the slow-cooking beans and plasma membrane, which was associated with the downregulated genes and thus important to the fast-cooking bean. In the GO term hierarchy, the plasma membrane (GO:0005886) is part of the cell periphery (GO:0071944), which describes any part of the cell that encompasses the cell cortex, plasma membrane, and any other encapsulating structures. These cell-wall-related GO terms are significant as they may provide insight into genes involved in the functioning of the cell wall, which is already implicated in the HTC defect in beans.

### 3.8. Functional Enrichment Analysis of Differentially Expressed Genes Identified between Fast- and Slow-Cooking Bean Varieties 

**Figure 8:**
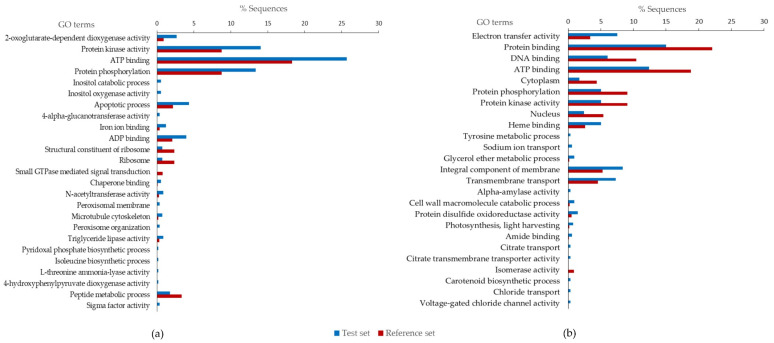
Fisher’s exact test enrichment plots showing the percentage of gene sequences that were enriched in a test set (up- or downregulated genes) against a reference set (annotated list of *P. vulgaris* genes retrieved from *P. vulgaris*_442_v2.1 genome). Genes that were upregulated in the slow-cooking bean (Pinto) represented the test set in (**a**), while the downregulated genes represented the test set in (**b**). DEGs with a log2 fold change of <−1 and >1 were selected for this analysis. The GO terms were ranked in order of most significantly to least significantly enriched sequences (*p* < 0.05).

To identify enriched gene sets, a list of DEGs with a log2 fold change of <−1 and >1 was selected for a gene-enrichment analysis using Fisher’s exact test [60]. Enriched genes are statistically over-represented or under-represented when compared to previously annotated genes (reference set), normally presented in groups exhibiting functional similarity. Accordingly, a list of 14910 genes was retrieved from the annotated common bean genome (*P. vulgaris*_442_v2.1) available in the Phytozome database and used as a reference set for this analysis [42]. Figure 8 displays the frequency of GO terms that were enriched in the upregulated and downregulated genes. A total of 25 most specific GO terms associated with the upregulated genes of the slow-cooking beans are presented in Figure 8a. Out of these, 21 GO terms were over-represented; i.e., they were significantly more abundant compared to the *P. vulgaris* reference set of annotated genes. Conversely, four GO terms were under-represented in the upregulated genes (test set) compared to the reference set (Figure 8a). The ATP binding GO term comprised the highest number of enriched genes (25.7% in the upregulated genes and 18.3% in the *P. vulgaris* reference set), indicating its importance in the slow-cooking bean variety. Protein kinase activity and protein phosphorylation GO terms were also important in the slow-cooking bean, having more genes in the slow-cooking bean compared to the *P. vulgaris* annotated reference set. The most significant GO term was 2-oxoglutarate-dependent dioxygenase activity (*p*-value 0.0004). Enzymes within this group constitute the second largest family of enzymes in plants, and are involved in numerous functions [61]. Important GO terms with a more direct link to the HTC defect included the inositol catabolic process and inositol oxygenase activity (both with a *p*-value of 0.003). Enzymes within these categories function in the metabolism of inositol and are sub-units of phytate, a compound that has been previously linked to the HTC defect [22]. All statistics related to significantly enriched GO terms for upregulated genes are shown in supplementary Table S4.

Further, 47 most specific GO functional groups were enriched in the downregulated gene pool (shown in Supplementary Table S5). Out of these, 38 GO terms were over-represented and 9 were under-represented in the downregulated genes (test set) com-pared to the *P. vulgaris* reference set. Figure 8b shows the top 25 GO terms in which electron transfer activity was the most significant, comprising 7.3% of the genes in the test set and 3.3% in the reference set (*p*-value = 0.00003). According to the GO database, the electron-transfer activity represents any molecular entity that serves as an electron acceptor and electron donor in an electron-transport chain [62]. Numerous cell functions are attached to this group, including the generation of a transmembrane electrochemical gradient. Heme binding, integral component of membrane, and transmembrane transport were GO terms that were also important in the downregulated genes, being over-represented in the fast-cooking bean compared to the *P. vulgaris* reference set. However, some of these enriched GO terms may not possess a direct link to the HTC defect. 

The cell wall macromolecule catabolic process (GO ID: 0016998) is, however, a critical biological process related to the HTC defect. This GO term defines the chemical reactions and pathways resulting in the breakdown of macromolecules that form part of a cell wall. It was significantly (*p* < 0.01) over-represented in the downregulated genes important to the fast-cooking bean, and consisted five 5 (0.9%) genes. Increased activity of enzymes that break down the cell wall would be ideal in the fast-cooking bean variety, as this action would result in softer seeds. Alpha-amylase activity (GO ID: 0004556) was also significantly (*p* < 0.01) enriched in the fast-cooking bean variety; it is involved in the hydrolysis of 1,4-alpha-d-glucosidic linkages in different polysaccharides in the cell, particularly starch molecules [63]. An increase in the activity of this enzyme would be equally important to achieve a fast-cooking bean phenotype due to improved gelatinization of starch.

### 3.9. Gene-Expression Patterns of Individual DEGs of the Fast- and Slow-Cooking Bean Varieties 

**Figure 9:**
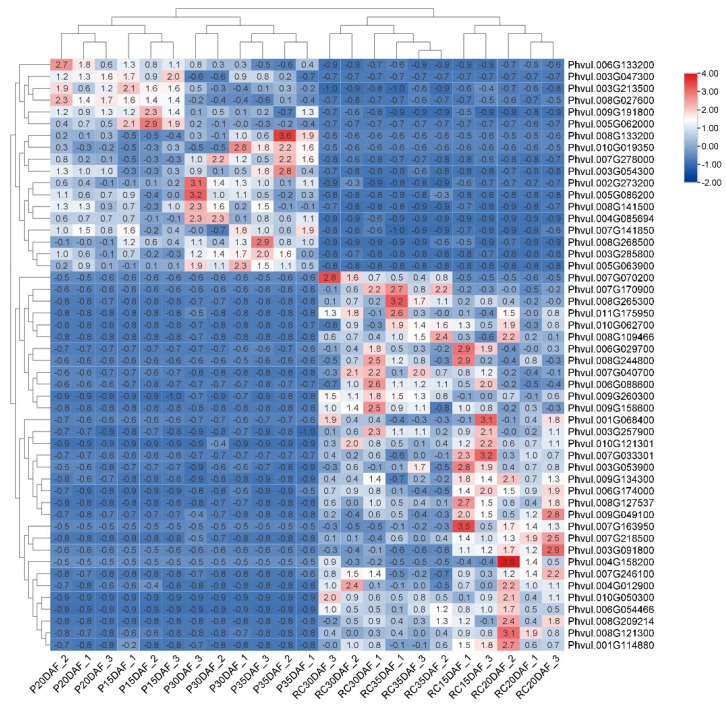
Heat map showing the expression patterns of top 50 DEGs identified between slow-cooking (Pinto) and fast-cooking (Rosecoco) common bean varieties. The gene-expression values used to construct the heat map were normalized using the counts per million (CPM) normalization method, and were further transformed into a logarithmic scale (shown within each cell). Normalized scaling was applied to rows, and the intensity of colour associated with DEGs ranges from red (upregulated genes) to blue (downregulated genes). The gene identifiers (IDs) are shown on the right side of the figure. Significant DEGs with an FDR value of <0.05 were selected to construct the heat map. Average clustering was performed between the rows and columns using Euclidean distance.

The order of these genes was similar to those shown in the heat map in Figure 9. Gene identifiers and descriptions were obtained from the PhytoMine tool available in the Phytozome database [35,42]. Thirteen genes that did not have any known description were omitted.

A clustered heat map revealed distinct patterns of expression of the top 50 significant genes (FDR < 0.05) genes, with sets of genes being expressed in either the fast- or slow-cooking variety at discrete development stages (Figure 9). A total of 18 genes were upregulated and 32 genes were downregulated in the slow-cooking bean at all sampled seed-development stages. Data mining performed on the Phytozome database [42] revealed interesting descriptions of the functional annotations associated with these genes, with only 37 of these genes being annotated (Table 2). The log2 fold change of these top 50 selected genes ranged from 9.6 to −6.5, representing a plant PEC family metallothionein gene (*Phvul.008g133200*) and an aspartyl protease-like protein (*Phvul.007g170900*) at both ends of the spectrum. A complete list of all the top 50 significant DEGs, including their log2 fold changes and log CPM values, are shown in supplementary Table S6.

The order of these genes was similar to those shown in the heat map in Figure 9. Gene identifiers and descriptions were obtained from the PhytoMine tool available in the Phytozome database [35,42]. Thirteen genes that did not have any known description were omitted.

Since the HTC defect has previously been linked to alterations that occur in the cell wall, identification of DEGs that are related to the functioning of the cell wall would be a valuable addition to HTC defect research. A general functional annotation of the top 50 DEGs using the Interpro (v85.0) and PFAM (v34.0) databases was therefore useful in identifying cell-wall-related genes [64,65]. The upregulated genes in the slow-cooking bean that were related to the functioning of the cell wall included *Phvul.009g191800* and *Phvul.003g054300* (log2 fold change of 3.8 and 4.9, respectively). *Phvul.009g191800* encodes a glycosylphosphatidylinositol (GPI) mannosyltransferase 2 involved in the GPI anchor biosynthetic process. GPI is a glycolipid that anchors different types of proteins to the plasma membrane (InterPro ID: IPR007315). *Phvul.003g054300* encodes a xyloglucan O-acetyltransferase, and belongs to the PC-esterase and trichome birefringence-like families. PC-esterases may modify plant cell wall properties via O-acetylation of cell wall polysaccharides including pectin and hemicellulose (InterPro ID: IPR026057/IPR029962/IPR025846). The genes related to the cell wall that were downregulated in the slow-cooking bean and therefore important to the fast-cooking bean included *Phvul.007g218500* and *Phvul.008g121300* (log2 fold change of −4.4 and −4.6, respectively). *Phvul.007g218500* encodes a pectinesterase/pectinesterase inhibitor 39-related whose function is hydrolysis of cell-wall-bound pectin (Pfam ID: PF01095). Finally, *Phvul.008g121300* encodes a callose synthase 3 involved in 1,3-beta-glucan synthesis (Pfam ID: PF14288). Altogether, these genes could be essential in the HTC phenomenon, which is predominantly associated with changes that occur in the plant cell wall.

### 3.10. Validation of the RNA Sequencing Data Using qRT-PCR of Potentially Significant Genes

**Figure 10:**
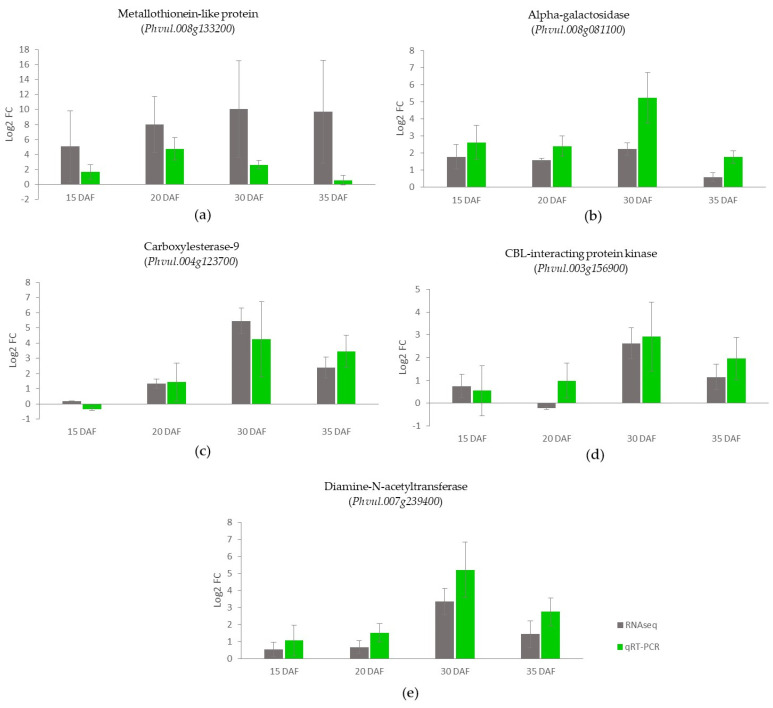
Comparison of RNA sequencing and qRT-PCR methods for analyzing the gene expression in the slow-cooking bean (Pinto) against the fast-cooking bean (Rosecoco). The bar graphs (**a**–**e**) show the gene expression of selected highly expressed genes in the slow-cooking bean. Gene-expression values are presented with log2 fold change (Log2FC) normalization. *B-tubulin* was used as the housekeeping gene for gene-expression normalization [53]. Error bars represent the standard error within samples.

Five interesting genes, namely *Phvul.008G133200*, *Phvul.007G239400*, *Phvul.008G081100*, *Phvul.003G156900,* and *Phvul.004G123700,* were selected for validation of the RNA sequencing data using qRT-PCR. These were DEGs that were highly expressed in the slow-cooking bean compared to the fast-cooking bean. The gene-expression patterns observed for these genes were generally similar in both methods, with a Pearson’s correlation coefficient of 0.71 (Figure 10). The first selected gene, *Phvul.008G133200*, showed the highest log2 fold change of 9.6 in the differential analysis between the slow- and fast-cooking beans (Table 2 and Figure 10a). It encodes a metallothionein-like protein that plays a role in storing and distributing zinc ions in seeds. *Phvul.008g081100* encodes an alpha-galactosidase that hydrolyses the terminal alpha-galactosyl groups from glycolipids and glycoproteins. It was the most-expressed gene among the genes within the cell periphery category (Figure 7 and Figure 10b). Among the hydrolases, *Phvul.004G123700* was most highly expressed. It encodes a hydrolase of the carboxylesterase-9 gene family that hydrolyses carboxylic esters (Figure 7 and Figure 10c). *Phvul.003G156900* (Figure 10d) encodes a CBL-interacting serine/threonine-protein kinase from the transferase and macromolecule modification categories, respectively, and *Phvul.007G239400* encodes a diamine-N-acetyltransferase (Figure 10e).

## 4. Discussion

The HTC defect is characterized by difficulty in realizing cell separation during cooking. The middle lamella is a crucial cell wall component that promotes cell adhesion. Found between two cells, the middle lamella is rich in pectin, the levels and chemical modification of which are crucial to regulating cell adhesion [66]. Typically, newly synthesized pectin is highly methyl-esterified and thus more fluid. The activity of a cell-wall-modifying protein, pectin methyl-esterase (PME), removes the methyl groups of pectin. De-methyl-esterified pectin is readily cross-linked by calcium, leading to a stiffer material and altering the mechanical properties of the cell wall [67,68]. This occurrence is the basis of the widely accepted pectin-cation-phytate theory of the development of the HTC defect. This theory postulates that during the storage of beans at a high temperature and relative humidity, the PME enzyme is produced, catalyzing the de-methyl-esterification of pectin [12]. A phytase enzyme further promotes the development of the HTC defect by the hydrolysis of phytates that are usually bound to divalent cations [22]. The divalent cations (in this case, calcium ions) migrate into the middle lamella and are bound to the de-methyl-esterified pectin, resulting in an insoluble, stiff, three-dimensional pectate network in the cell wall and middle lamella [12,22].

We first explored the activity of the PME enzyme in *P. vulgaris* using an enzymatic assay, which revealed that the PME activity was slightly higher in the slow-cooking bean, especially in the early stages of development of the seed. However, there was no detectable difference between the varieties at later stages of seed development (Figure 1). The higher activity of PME in the slow-cooking bean observed at the early stages of seed development was in agreement with the pectin-cation-phytate theory, since it would promote the increased formation of the stiff pectate-cation structure. However, the lack of apparent differences in PME activity during the later stages of development prompted an in-depth study to identify more conclusive evidence of the contribution of the PME enzyme to the HTC defect. For this PME enzymatic assay, the crude protein extract was utilized as a source of PME for the hydrolysis of pectin, and as such, was not specific to the gene level, making it a less-discriminative assay for an enzyme encoded by multiple genes.

Typically, PME/PMEI genes belong to large multigene families in many plant species. For instance, 67 and 81 PME genes were identified in *Arabidopsis thaliana* and *Pyrus bretschneideri* (pear), respectively; while in flax seed, 105 PMEs and 95 PMEIs were identified [69,70]. We identified several PME-related genes and their isoforms in *P. vulgaris*; a total of 74 putative PMEs and 39 putative PMEIs (Figure 2). Genes from each of the two families in *P. vulgaris* showed a striking difference in their exon–intron structure. While PME genes are long in size and have multiple exons and introns, most PMEIs consist of a single exon gene with no introns, and are thus remarkably shorter in length (Figure 4). Whereas exons are indisputably known for their function of coding for mRNA and subsequent proteins, the role of introns is less acknowledged. It may include many functions, such as coding for sequences that regulate transcription; for instance, the untranslated RNAs or splicing control elements [71,72,73]. Thus, gene-sequence conservation is high in exon regions, whereas introns are believed to evolve under natural selection [73,74]. The exon size is dictated mainly by the inherently coded proteins. However, intron size may be influenced by various factors that control gene expressions, such as the presence of regulatory elements and alternative splicing, as well as other unexplained factors such as insertion of transposable elements and frequency and size of deletions, among others [73].

Despite their variety, all PMEs are known to catalyze the same reaction, consistent with the pectin-cation-phytate theory described earlier. In the detailed study of PME-related genes, we established that the PME gene *Phvul. 010g080300* was one of the most highly expressed PMEs in fast- and slow-cooking beans (Figure 3 and Figure 5). However, this PME gene was more significantly expressed in the fast-cooking bean, and more so at the late stage of seed development (30 and 35 DAF). Out of the top 50 DEGs ranked by FDR significance, we only identified one PME-related gene; i.e., *Phvul.007g218500,* which was downregulated in the HTC bean with a log fold change of −4.4 (Figure 9 and Table 2). This observation was contrary to what was expected concerning the pectin-cation-phytate theory, which implies that PMEs will be highly expressed in a slow-cooking bean. We further explored the expression of PMEs in *P. vulgaris* using qRT-PCR to validate these results. It was apparent that the expression of the specific PME *Phvul.010g080300* was higher in the seeds of the fast-cooking bean as determined by qRT-PCR.

This deviation from the expected results can be explained, as suggested by Koch and Nevins [75], that the de-methyl-esterification caused by PMEs may render pectin susceptible to degradation by other pectinolytic enzymes, further promoting cell separation [75]. This hypothesis would exclude the pectate–calcium ion linkages that occur after PME activity and may cause the fast-cooking phenotype seen in our bean. Chigwedere et al. [15] observed no significant difference in the degree of methyl-esterification (DM) of chelator-extractable pectin between fresh and aged bean samples, similarly suggesting that cation-mediated cross-linking was not crucial in ageing-induced hardening of beans. Further, Bosch and Hepler [76] suggested that protons released during the de-methyl-esterification process can cause a localized reduction in pH, which can either inhibit activity of PME enzymes [77] or further stimulate the activity of cell wall hydrolases such as polygalacturonases and pectate lyases [78]. The enzymatic actions of these hydrolases result in the loosening of the cell wall in fast-cooking beans. As such, it may be essential to characterize the activity of these enzymes and their functional products in fast- and slow-cooking beans to determine their influence on the HTC defect [79].

The action of PMEs can be countered by the activity of pectin methyl-esterase inhibitors (PMEIs), and as such, the balance of these two proteins and their activities have effects on the mechanical properties of the middle lamella, and must be incorporated into the global overview of the development of the HTC defect. Concerning the well-accepted pectin-hardening theory, a high activity of the PME inhibitors may well result in the decreased activity of PMEs, resulting in the HTC phenotypic trait. Compared to other PMEIs and all PME-related genes in general, *Phvul.005g007600* showed a remarkably high expression, alluding to its essential role in regulating PMEs and pectin modification (Figure 3 and Figure 5).

Complete transcriptome analysis also allowed us to identify several DEGs, thus providing a valuable gene pool for the identification of more genes that may contribute to the HTC defect (Figure 6). For example, other components of the cell wall, mainly cellulose, hemicellulose, and lignin, are known to affect the rigidity of the cell wall, and may contribute to additional difficulty in achieving cell separation during cooking. We identified three other genes among the top 50 DEGs related to the cell wall (Figure 9 and Table 2). *Phvul.009g191800* encodes a glycosylphosphatidylinositol (GPI) mannosyltransferase 2 enzyme involved in the biosynthesis of a complex glycolipid called phosphatidylinositol glycan class V (PIGV). The glycolipid functions as a membrane anchor for many proteins, and plays a role in multiple cellular processes, including protein sorting and signal transduction. The attachment of GPI is a common post-translational modification for anchoring proteins to the outer surface of the plasma membrane in eukaryotes, and is thus useful for the proper functioning of membrane proteins [80]. *Phvul.003g054300* encodes a xyloglucan O-acetyltransferase that is involved in the synthesis of a protein-altered xyloglucan 4 (AXY4) and belongs to the PC-esterase and trichome birefringence-like (TBL) families. The PC-esterase family contains the powdery mildew resistance protein (PMR5) and Cas1p domains involved in modifying the plant cell wall properties via O-acetylation of cell wall polysaccharides, including hemicelluloses, pectic polysaccharides, and lignin [81,82]. O-acetylation impacts the rheological properties and hinders the enzymatic breakdown of the cell wall polysaccharides through steric hindrance and conformational changes to the polysaccharides [81]. TBLs have been reported to contribute to the biosynthesis and deposition of cell wall cellulose, and one TBL gene was identified as a potential candidate that influenced cooking time in a GWAS study by Cichy et al. [20,83]. *Phvul.009g191800* and *Phvul.003g054300* were both upregulated in the slow-cooking Pinto bean, and may contribute to the increased cooking time associated with HTC beans. *Phvul.008g121300* encodes a callose synthase 3 (1,3-beta-glucan) enzyme that was downregulated in the slow-cooking Pinto bean. Callose is a cell wall polymer involved in several fundamental biological processes, ranging from plant development to responses to abiotic and biotic stresses. The biosynthesis and degradation of callose in the plasmodesmata help regulate permeability during abiotic and biotic stresses [84,85]. The regulation of this gene may also allow for cell wall permeability required for the fast-cooking bean [86].

Several GO groups were represented in the upregulated and downregulated sequences (Figure 7). These did not openly highlight essential functions that may be specific to cooking time. However, the functional classification of DEGs upregulated within the cellular compartment presented genes that were upregulated within the cell periphery. Individual analysis of these genes may reveal important candidate genes. Further, examining the enriched GO terms revealed that there were genes involved in the inositol catabolic process and inositol oxygenase activity that were enriched in the slow-cooking bean (Figure 8). Phytates are complexes of inositol that are attached to about four to six phosphate groups. The catabolism of phytates has already been linked to the HTC defect as described in the phytate-phytase-pectin theory. Inositol oxygenase is the key enzyme in converting myo-inositol into UDP-glucuronic acid, the primary precursor of many cell wall polysaccharides [87]. Sequences enriched within the inositol catabolic process and inositol oxygenase activity may have a profound effect on the HTC defect. On the other hand, the cell wall macromolecule catabolic process was enriched in the downregulated sequences. In general, this GO term describes chemical reactions and pathways that result in the breakdown of macromolecules that form part of a cell wall. This action may be useful in promoting ease in the separation of the fast-cooking beans’ cell walls during cooking.

Further to the information gained from this study, we recommend integrating multiple ‘omics’ techniques such as proteomics and metabolomics that will provide additional phenotypic measurements to which transcriptomic data can be anchored. Previous studies that have measured metabolites related to the HTC defect, such as pectin, phytates, starch, and minerals, have utilized precise techniques with low-throughput data and associations [15,25,88,89]. Beans with the HTC defect were found to contain a high amount of chelator-extractable pectins compared to water-extractable pectins, consistent with the HTC defect [25]. Chigwedere et al. [88] additionally demonstrated a secondary role of starch in influencing the HTC defect, possibly due to the cell-wall-restricted uncoiling of gelatinized starch into a viscous gel, promoting its retrogradation. In our study, alpha-amylase activity was among the significantly enriched processes in the fast-cooking bean, and could promote starch gelatinization and bean softening. Characterizing the activity of crucial enzymes identified in this study, such as xyloglucan O-acetyltransferase, will further provide new insights into the occurrence of the HTC defect in beans.

Nonetheless, the integration of transcriptomic and metabolomic data may fail to result in direct associations between metabolites and transcripts because it is impossible to link each transcript to a metabolite [90]. As such, complex statistical techniques must be employed to detect inherent relationships in the data set. On the contrary, integrated transcriptomics and proteomics data may allow for the mapping of transcripts to single proteins, resulting in similar profiles, even though there can still be significant deviations in the amount of transcript and translated proteins [91].

## 5. Conclusions

The pectin methyl-esterase (PME) enzyme and cell wall components have been widely implicated in the development of the hard-to-cook (HTC) defect using bioprocessing techniques. The transcriptomics approach utilized in this study revealed that multiple genes coding for PME and its inhibitor (PMEI) exist and are variably expressed in the common bean. This finding proved that the HTC defect is a complex phenomenon that involves an interaction of multiple molecules. Nevertheless, highly expressed genes encoding PMEs and PMEIs were identified as having a potential contribution to the HTC defect. Overall, PME-encoding genes were expressed highly in the fast-cooking bean, while their inhibitors were expressed more in the slow-cooking bean variety. Additionally, numerous differentially expressed genes (DEGs) were identified between the fast- and slow-cooking bean varieties at different stages of seed development, providing a much-needed gene pool that will be useful in identifying subsidiary candidate genes of the hard-to-cook defect. Four genes associated with structural alterations within the cell wall were among the highly ranked DEGs. This study thus established the dynamic involvement of the PME-related genes and the cell wall in the occurrence of the HTC defect.

## Figures and Tables

**Table 1:** Quality-control statistics from an analysis of RNA sequencing raw read results.

Description	Minimum	Maximum	Mean	Total
Number of libraries	-	-	-	24
Number of mapped reads	3,735,119	10,139,058	5,890,219	141,365,247
Number of alignments	5,359,638	29,044,091	10,469,383	251,265,193
Number of input reads (per library)	3,922,953	11,186,658	6,209,020	149,016,471
Average input read length	43	49	47.71	-
Uniquely mapped reads	3,288,861	8,020,182	4,972,255	119,334,111
Uniquely mapped reads (%)	61.16	90.58	80.82	-
Average mapped length	41.45	48.85	47.44	-
Total number of counts	2,878,112	7,374,463	4,431,196	106,348,694

**Table 2:** Description of top 50 DEGs with a distinct expression pattern in the slow-cooking (Pinto) and fast-cooking (Rosecoco) bean varieties.

Gene Identifier	Log2 Fold Change	Gene Description
*Phvul.006g133200*	3.712249	Leucine-rich repeat protein kinase-like protein
*Phvul.003g047300*	6.649919	Cadmium/zinc-transporting ATPase HMA1, chloroplastic-related
*Phvul.003g213500*	2.991666	Homeobox protein knotted-1-like 7
*Phvul.008g027600*	3.377875	Glutathione s-transferase kappa
*Phvul.009g191800*	3.820829	GPI mannosyltransferase; Phosphatidylinositol glycan, class V
*Phvul.008g133200*	9.62583	Plant PEC family metallothionein (Metallothio_PEC)
*Phvul.010g019350*	7.117989	Small EDRK-rich factor 1
*Phvul.007g278000*	4.789283	Expressed protein-related
*Phvul.003g054300*	4.895153	Xyloglucan O-acetyltransferase; protein altered xyloglucan 4
*Phvul.002g273200*	3.185114	Histone deacetylase 6
*Phvul.005g086200*	4.975348	Pre-mRNA-processing factor 8 (PRPF8, PRP8)
*Phvul.008g141500*	3.561553	Chromatin remodelling complex WSTF-ISWI, small subunit
*Phvul.004g085694*	4.860125	Androgen induced inhibitor of proliferation AS3/PDS5-related
*Phvul.008g268500*	4.704867	Lob domain-containing protein 23-related
*Phvul.005g063900*	8.368925	11-oxo-beta-amyrin 30-oxidase/CYP72A154
*Phvul.007g070200*	−7.59207	Copper transport protein atox1-related
*Phvul.007g170900*	−6.47656	Aspartyl protease-like protein
*Phvul.008g265300*	−5.53117	Genomic DNA, chromosome 3, p1 clone: MSD21
*Phvul.006g029700*	−4.7696	Protein RCC2
*Phvul.008g244800*	−5.32578	Leucine-rich repeat protein kinase-like protein
*Phvul.007g040700*	−4.07947	S-methyl-5-thioribose kinase/MTR kinase
*Phvul.006g088600*	−3.57527	Alpha/beta-hydrolases superfamily protein
*Phvul.009g260300*	−3.06147	Phosphatase DCR2-related
*Phvul.009g158600*	−5.9323	SAUR family protein (SAUR)
*Phvul.001g068400*	−4.71518	PPR repeat (PPR)/(PPR_1)/PPR repeat family (PPR_2)
*Phvul.003g257900*	−2.5961	Alpha/beta hydrolase fold-containing protein
*Phvul.010g121301*	−4.62911	Ribosome production factor 1
*Phvul.007g033301*	−5.91743	H/ACA ribonucleoprotein complex subunit 3 (NOP10, NOLA3)
*Phvul.003g053900*	−2.58937	Gluconokinase (E2.7.1.12, gntk, idnk)
*Phvul.009g134300*	−3.70006	Bidirectional sugar transporter sweet1
*Phvul.008g127537*	−5.3512	Lysosomal acid lipase-related
*Phvul.009g049100*	−4.32872	6-phosphogluconolactonase
*Phvul.007g218500*	−4.36224	Pectinesterase/pectinesterase inhibitor 39-related
*Phvul.003g091800*	−3.99388	Aluminum induced protein with YGL and LRDR motifs
*Phvul.004g158200*	−7.748	Legume lectin domain (Lectin_legb)
*Phvul.004g012900*	−3.83536	Leucine-rich repeat-containing protein
*Phvul.008g121300*	−4.59491	Callose synthase 3

## Data Availability

The original RNA sequencing data sets presented in this study are openly available in the European Nucleotide Archive database (https://www.ebi.ac.uk/ena/browser/view/PRJEB45523?show=reads, accessed on 23 May 2022) under the accession number PRJEB45523.

## Supplementary Materials

The following supporting information can be downloaded at: https://www.mdpi.com/article/10.3390/foods11121692/s1, Figure S1: Heat map showing the expression of genes encoding pectin methyl-esterases (PMEs) and pectin methyl-esterase inhibitors (PMEIs) in a slow-cooking (Pinto) and a fast-cooking (Rosecoco) *P. vulgaris* variety, Figure S2: Exon–intron structure of genes encoding the pectin methyl-esterase and pectin methyl-esterase inhibitors in *P. vulgaris*, Table S1: Primers used for qRT-PCR assay, Table S2: Functional characterization and molecular properties of genes encoding pectin methyl-esterases (PMEs) and pectin methyl-esterase inhibitors (PMEIs) in *P. vulgaris*, Table S3: Mapping statistics of RNA libraries from a single-end RNA sequencing study of slow-cooking and fast-cooking bean varieties, Table S4: Enriched GO terms associated with upregulated sequences identified using Fisher’s exact test, Table S5: Enriched GO terms associated with downregulated sequences identified using Fisher’s exact test, Table S6: Gene list of the top 50 differentially expressed genes (DEGs) with a distinct expression pattern in the slow-cooking (Pinto) and fast-cooking (Rosecoco) bean varieties. Reference [92] is cited in the supplementary materials.
