# Supplementary material for: Transcriptome-Guided Identification of Pectin Methyl-Esterase-Related Enzymes and Novel Molecular Processes Effectuating the Hard-to-Cook Defect in Common Bean (Phaseolus vulgaris L.)
Source: Foods. 2022 Jun 9;11(12):1692. doi: 10.3390/foods11121692 (PMC9222787; doi:10.3390/foods11121692)
Supplement: Supplementary file 1 [file foods-11-01692-s001.zip › foods-1724433-supplementary.pdf]

**Table S1.** Primers used for qRT-PCR assay.

| Gene Identifier | Forward primer (5′- 3′) | Tm (°C) | Reverse primer (5′- 3′) | Tm (°C) | Amplicon length |
|---|---|---|---|---|---|
| *Phvul.008G133200* | GAGGGATGGCAGCTTGTGAT | 60.11 | TCTTGGAACTGAAGAGAT-TCGGG | 60.06 | 145 |
| *Phvul.008g081100* | CTCCTCCAATGGGGTGGAAC | 60.03 | GAGCCATCGATAAAGCCCGA | 59.97 | 438 |
| *Phvul.004G123700* | GTCTTTTCCGCTGTGCGTTT | 59.97 | ACACCCTCCACCCTAGATCC | 60.03 | 143 |
| *Phvul.003G156900* | TGCCGAGAAAGGTGGAA-GAC | 59.97 | CAAACAGGGGCGACAAATCG | 60.11 | 123 |
| *Phvul.007G239400* | CGAGTGGAGGATTTGCAGGT | 60.04 | ACCCATTCAAAC-CTTCCCTAACA | 59.86 | 154 |
| *Phvul.010g080300* | GCAAATT-GGACAGAAACTGC | 59.98 | TTGAGTCTTACCAAACGTGG | 59.55 | 130 |
| *Phvul.005g07600* | GCAACAAGTTCAAGGGCCTG | 59.97 | ATGTGCCAGTCGAAGTCCTG | 60.04 | 148 |
| *B-tubulin* | CCGTTGTGGAGCCTTACAAT | 62.40 | GCTTGGGGTCCTGAAACAA | 62.40 | 117 |

**Table S2.** Functional characterization and molecular properties of genes encoding pectin methyl-esterases (PMEs) and pectin methyl-esterase inhibitors (PMEIs) in *P. vulgaris.*

| No. | Gene ID | pI | Mw (kDa) | Function |
|---|---|---|---|---|
| 1 | *Phvul.005G007800* | 9.05 | 59859.9 | Probable pectinesterase/pectinesterase inhibitor |
| 2 | *Phvul.011G005000* | 9.79 | 59990.6 | Probable pectinesterase/pectinesterase inhibitor |
| 3 | *Phvul.010G079900* | 8.83 | 57085.1 | Pectinesterase/pectinesterase inhibitor |
| 4 | *Phvul.010G080000* | 7.96 | 43093.5 | Pectinesterase superfamily |
| 5 | *Phvul.002G318500* | 9.21 | 37891.5 | Pectinesterase |
| 6 | *Phvul.010G080300* | 7.62 | 53601.6 | Pectinesterase family protein |
| 7 | *Phvul.001G209400* | 8.71 | 62133.6 | Probable pectinesterase/pectinesterase inhibitor |
| 8 | *Phvul.007G218500* | 9.13 | 61741.3 | Probable pectinerase/ pectinesterase inhibitor |
| 9 | *Phvul.009G261700* | 9.35 | 63596.1 | Pectinesterase/pectinesterase inhibitor |
| 10 | *Phvul.010G123066* | 8.53 | 66582.0 | Pectinesterase/pectinesterase inhibitor |
| 11 | *Phvul.008G158900* | 9.37 | 66149.9 | Probable pectinesterase/pectinesterase inhibitor |
| 12 | *Phvul.009G053000* | 9.1 | 63839.3 | Probable pectinesterase/pectinesterase inhibitor |
| 13 | *Phvul.002G048400* | 6.4 | 60809.9 | Pectinesterase/pectinesterase inhibitor |
| 14 | *Phvul.002G048700* | 7.61 | 60345.5 | Pectinesterase/pectinesterase inhibitor |
| 15 | *Phvul.002G048500* | 7.97 | 60899.0 | Pectinesterase/pectinesterase inhibitor |
| 16 | *Phvul.002G048600* | 8.2 | 61088.2 | Pectinesterase/pectinesterase inhibitor |
| 17 | *Phvul.006G088000* | 8.05 | 60665.8 | Putative pectinesterase/pectinesterase inhibitor |
| 18 | *Phvul.009G222300* | 6.47 | 56428.1 | Probable pectinesterase/pectinesterase inhibitor |
| 19 | *Phvul.003G126500* | 7.03 | 56134.8 | Probable pectinesterase/pectinesterase inhibitor |
| 20 | *Phvul.009G222100* | 5.42 | 57370.5 | Probable pectinesterase/pectinesterase inhibitor |
| 21 | *Phvul.003G126600* | 6.23 | 58295.6 | Probable pectinesterase/pectinesterase inhibitor |
| 22 | *Phvul.009G222400* | 4.88 | 44987.2 | Probable pectinesterase/pectinesterase inhibitor |
| 23 | *Phvul.009G222500* | 5.08 | 59161.4 | Probable pectinesterase/pectinesterase inhibitor |
| 24 | *Phvul.005G011900* | 8.68 | 47938.2 | Probable pectinesterase/pectinesterase inhibitor; Provisional |
| 25 | *Phvul.011G188900* | 8.63 | 68119.0 | Probable pectinesterase/pectinesterase inhibitor; Provisional |
| 26 | *Phvul.006G088100* | 6.53 | 61738.0 | Probable pectinerase/ pectinesterase inhibitor |
| 27 | *Phvul.007G198200* | 5.38 | 58645.7 | Probable pectinerase/ pectinesterase inhibitor |
| 28 | *Phvul.001G209100* | 8.58 | 61495.4 | Probable pectinerase/ pectinesterase inhibitor |
| 29 | *Phvul.007G246700* | 9.06 | 62411.6 | Probable pectinerase/ pectinesterase inhibitor |
| 30 | *Phvul.001G209200* | 5.2 | 61410.0 | Probable pectinerase/ pectinesterase inhibitor |

| | | | | |
|---|---|---|---|---|
| 31 | *Phvul.001G209300* | 5.67 | 61794.2 | Probable pectinerase/ pectinesterase inhibitor |
| 32 | *Phvul.005G184400* | 7.64 | 39386.6 | Pectinesterase superfamily |
| 33 | *Phvul.009G222700* | 8.5 | 58305.1 | Probable pectinesterase/pectinesterase inhibitor |
| 34 | *Phvul.005G012000* | 9.3 | 63452.1 | Putative pectinesterase/pectinesterase inhibitor |
| 35 | *Phvul.011G189000* | 8.69 | 50206.2 | Putative pectinesterase/pectinesterase inhibitor |
| 36 | *Phvul.010G123132* | 6.1 | 63587.8 | Pectinesterase |
| 37 | *Phvul.001G217300* | 6.29 | 62800.1 | Putative pectinesterase/pectinesterase inhibitor |
| 38 | *Phvul.001G217400* | 6.47 | 62740.1 | Putative pectinesterase/pectinesterase inhibitor |
| 39 | *Phvul.007G210400* | 7.97 | 61825.4 | Putative pectinesterase/pectinesterase inhibitor |
| 40 | *Phvul.007G210200* | 6.12 | 60734.0 | Putative pectinesterase/pectinesterase inhibitor |
| 41 | *Phvul.007G210300* | 5.31 | 62191.2 | Putative pectinesterase/pectinesterase inhibitor |
| 42 | *Phvul.003G126700* | 6.5 | 66884.4 | Putative pectinesterase/pectinesterase inhibitor |
| 43 | *Phvul.009G222600* | 5.04 | 69117.0 | Putative pectinesterase/pectinesterase inhibitor |
| 44 | *Phvul.003G107300* | 5.56 | 64688.3 | Probable pectinesterase/pectinesterase inhibitor |
| 45 | *Phvul.009G250600* | 7.89 | 63591.4 | Probable pectinesterase/pectinesterase inhibitor |
| 46 | *Phvul.006G088200* | 6.79 | 65156.2 | Probable pectinesterase/pectinesterase inhibitor |
| 47 | *Phvul.007G198100* | 9.26 | 64965.1 | Probable pectinesterase/pectinesterase inhibitor |
| 48 | *Phvul.005G103500* | 9.24 | 51045.7 | Probable pectinesterase/pectinesterase inhibitor |
| 49 | *Phvul.005G015000* | 6.97 | 65201.1 | Probable pectinesterase/pectinesterase inhibitor |
| 50 | *Phvul.005G104100* | 8.58 | 52214.6 | Probable pectinesterase/pectinesterase inhibitor |
| 51 | *Phvul.008G039000* | 6.18 | 35413.3 | Pectinesterase |
| 52 | *Phvul.008G271600* | 9.02 | 33810.3 | Pectinesterase superfamily |
| 53 | *Phvul.008G271800* | 8.32 | 35018.5 | Pectinesterase superfamily |
| 54 | *Phvul.008G271500* | 9.37 | 66149.9 | Pectinesterase superfamily |
| 55 | *Phvul.008G272000* | 5.06 | 37727.4 | Pectinesterase superfamily |
| 56 | *Phvul.007G201900* | 6.57 | 35029.6 | Pectinesterase superfamily |
| 57 | *Phvul.007G202000* | 8.33 | 36440.4 | Pectinesterase superfamily |
| 58 | *Phvul.003G015600* | 8.41 | 37627.5 | Pectinesterase superfamily |
| 59 | *Phvul.003G015700* | 7.72 | 37851.1 | Pectinesterase superfamily |
| 60 | *Phvul.006G118422* | 8.16 | 38617.6 | Pectinesterase superfamily |
| 61 | *Phvul.008G277900* | 5 | 40102.0 | Pectinesterase superfamily |
| 62 | *Phvul.008G278000* | 8.96 | 41858.4 | Pectinesterase superfamily |
| 63 | *Phvul.003G027000* | 6.46 | 40920.4 | Pectinesterase family protein |
| 64 | *Phvul.003G127700* | 9.36 | 37962.0 | Pectinesterase family protein |
| 65 | *Phvul.007G212000* | 9.27 | 41019.5 | Pectinesterase superfamily |
| 66 | *Phvul.002G308000* | 9.23 | 41195.1 | Pectinesterase |
| 67 | *Phvul.004G175200* | 8.11 | 41993.8 | Pectinesterase |
| 68 | *Phvul.001G140600* | 6.64 | 42945.3 | Probable pectinesterase |
| 69 | *Phvul.001G183100* | 6.12 | 42838.8 | Probable pectinesterase |
| 70 | *Phvul.002G063100* | 8.9 | 39525.5 | Pectinesterase superfamily |
| 71 | *Phvul.003G220500* | 8.77 | 38814.0 | Pectinesterase superfamily |
| 72 | *Phvul.002G270900* | 6.02 | 58906.4 | Probable pectinesterase/pectinesterase inhibitor |
| 73 | *Phvul.009G148900* | 8.85 | 60976.1 | Probable pectinesterase/pectinesterase inhibitor |
| 74 | *Phvul.002G060750* | 9.63 | 22179.5 | PMEI-like_3 |
| 75 | *Phvul.003G217200* | 9.27 | 22898.4 | PMEI-like_3 |
| 76 | *Phvul.005G007600* | 8.6 | 20505.7 | PMEI-like_3 |
| 77 | *Phvul.011G005200* | 7.7 | 22252.4 | PMEI-like_3 |
| 78 | *Phvul.011G005300* | 6.42 | 22044.1 | PMEI-like_3 |
| 79 | *Phvul.003G128500* | 9.96 | 21779.8 | PMEI-like_3 |
| 80 | *Phvul.009G224200* | 11.12 | 23393.9 | PMEI-like_3 |
| 81 | *Phvul.002G318700* | 5.67 | 22696.5 | PMEI-like_3 |
| 82 | *Phvul.003G021400* | 5.31 | 22370.0 | PMEI-like_3 |
| 83 | *Phvul.003G021500* | 4.76 | 18144.3 | PMEI-like_3 |

| | | | | |
|---|---|---|---|---|
| 84 | *Phvul.001G207300* | 9.25 | 21608.8 | PMEI-like_3 |
| 85 | *Phvul.005G007700* | 5.25 | 22646.5 | PMEI-like_3 |
| 86 | *Phvul.011G005100* | 7.61 | 21751.9 | PMEI-like_3 |
| 87 | *Phvul.009G188700* | 7 | 24752.3 | Pectinesterase |
| 88 | *Phvul.002G179966* | 7.67 | 14812.2 | PMEI |
| 89 | *Phvul.002G180032* | 9.3 | 9133.4 | PMEI_like superfamily |
| 90 | *Phvul.010G080600* | 9.68 | 24450.2 | PMEI-like_3 |
| 91 | *Phvul.010G080700* | 9.73 | 24714.7 | PMEI |
| 92 | *Phvul.007G213550* | 6.94 | 24732.1 | PMEI-like_2 |
| 93 | *Phvul.007G213600* | 8.21 | 24693.1 | PMEI-like_2 |
| 94 | *Phvul.007G213500* | 4.81 | 21334.2 | PMEI-like_2 |
| 95 | *Phvul.001G213900* | 4.45 | 23764.9 | PMEI-like_2 |
| 96 | *Phvul.006G130500* | 6.3 | 22561.0 | PMEI-like_2 |
| 97 | *Phvul.006G130600* | 6.32 | 22623.1 | PMEI-like_2 |
| 98 | *Phvul.007G271600* | 5.05 | 18609.1 | PMEI |
| 99 | *Phvul.007G271650* | 5.73 | 21188.3 | PMEI |
| 100 | *Phvul.006G117500* | 7.3 | 33241.4 | PMEI-like_2 |
| 101 | *Phvul.003G227500* | 7.57 | 20624.6 | PMEI-Pla_a_1_like |
| 102 | *Phvul.006G071800* | 6.57 | 20351.2 | PMEI-Pla_a_1_like |
| 103 | *Phvul.010G041500* | 8.09 | 21169.2 | PMEI-Pla_a_1_like |
| 104 | *Phvul.003G227700* | 4.21 | 21006.1 | PMEI-Pla_a_1_like |
| 105 | *Phvul.006G119800* | 6.09 | 20397.7 | PMEI |
| 106 | *Phvul.009G149400* | 7.6 | 19738.7 | PME_inhibitor |
| 107 | *Phvul.007G246800* | 4.47 | 19899.8 | PMEI-like_1 |
| 108 | *Phvul.009G236000* | 4.59 | 19680.7 | PMEI_like superfamily |
| 109 | *Phvul.001G208900* | 4.4 | 20967.1 | PMEI_like superfamily |
| 110 | *Phvul.001G209000* | 4.49 | 20911.2 | PMEI_like superfamily |
| 111 | *Phvul.001G208600* | 4.94 | 22856.7 | PMEI_like superfamily |
| 112 | *Phvul.001G208700* | 4.58 | 20910.3 | PMEI_like superfamily |
| 113 | *Phvul.001G208800* | 4.68 | 21000.4 | PMEI_like superfamily |

Gene ID (gene identifier) and functions were retrieved from Phytozome [42] while the protein domains were identified using the NCBI CDD Batch Search online tool [38]; pI (theoretical isoelectric point) and Mw (molecular weight) were retrieved from ExPASy online tool (ExPASy - Compute pI/Mw tool).

**Table S3.** Mapping statistics of RNA libraries from a single end RNA sequencing study of slow-cooking and fast-cooking bean varieties.

| Sample | Number of mapped reads | Total number of alignments | Number of secondary alignments | Number of non-unique alignments | Aligned to genes | Ambiguous alignments | No feature assigned | Exonic reads | Intronic reads | Intergenic reads | Intronic/intergenic overlapping exon reads |
|---|---|---|---|---|---|---|---|---|---|---|---|
| **8084** | 5,159,992 | 10,823,251 | 5,663,259 | 5,896,777 | 3,107,134 | 1,320 | 1,136,958 | 3,107,134 / 73.21% | 221,023 / 5.21% | 915,935 / 21.58% | 178,099 / 4.2% |
| **8086** | 6,373,774 | 9,888,934 | 3,515,160 | 3,874,635 | 4,291,675 | 2,738 | 1,386,680 | 4,291,675 / 75.58% | 236,518 / 4.17% | 1,150,162 / 20.26% | 324,852 / 5.72% |
| **8090** | 6,423,261 | 11,303,085 | 4,879,824 | 5,147,517 | 4,524,265 | 2,879 | 1,062,868 | 4,524,265 / 80.98% | 196,451 / 3.52% | 866,417 / 15.51% | 257,821 / 4.61% |
| **8093** | 5,178,562 | 6,404,691 | 1,226,129 | 1,514,202 | 4,211,212 | 5,825 | 590,059 | 4,211,212 / 87.71% | 135,719 / 2.83% | 454,340 / 9.46% | 161,793 / 3.37% |
| **8095** | 3,735,119 | 8,681,611 | 4,946,492 | 5,388,093 | 2,210,014 | 2,597 | 558,416 | 2,210,014 / 79.83% | 143,298 / 5.18% | 415,118 / 14.99% | 91,965 / 3.32% |
| **8096** | 6,056,570 | 11,134,668 | 5,078,098 | 5,691,966 | 3,848,952 | 3,348 | 1,128,234 | 3,848,952 / 77.33% | 156,634 / 3.15% | 971,600 / 19.52% | 273,950 / 5.5% |
| **8097** | 7,198,371 | 10,196,347 | 2,997,976 | 3,677,913 | 5,476,171 | 7,328 | 808,685 | 5,476,171 / 87.13% | 193,833 / 3.08% | 614,852 / 9.78% | 194,468 / 3.09% |
| **8099** | 9,291,406 | 29,044,091 | 19,752,685 | 20,178,265 | 4,731,185 | 3,346 | 1,447,706 | 4,731,185 / 76.57% | 281,416 / 4.55% | 1,166,290 / 18.88% | 341,007 / 5.52% |

| | | | | | | | | | | | |
|---|---|---|---|---|---|---|---|---|---|---|---|
| **8101** | 4,824,073 | 9,271,130 | 4,447,057 | 4,999,276 | 3,267,566 | 4,855 | 563,442 | 3,267,566 / 85.29% | 151,618 / 3.96% | 411,824 / 10.75% | 133,692 / 3.49% |
| **8104** | 4,069,323 | 6,908,039 | 2,838,716 | 3,255,611 | 2,788,998 | 2,227 | 613,961 | 2,788,998 / 81.96% | 116,452 / 3.42% | 497,509 / 14.62% | 159,204 / 4.68% |
| **8106** | 10,139,058 | 22,622,776 | 12,483,718 | 13,285,782 | 5,887,647 | 4,771 | 2,066,525 | 5,887,647 / 74.02% | 356,991 / 4.49% | 1,709,534 / 21.49% | 548,253 / 6.89% |
| **8108** | 4,648,578 | 7,621,544 | 2,972,966 | 3,202,730 | 3,307,388 | 5,285 | 765,383 | 3,307,388 / 81.21% | 174,896 / 4.29% | 590,487 / 14.5% | 143,265 / 3.52% |
| **8110** | 9,686,527 | 14,890,498 | 5,203,971 | 6,607,681 | 6,924,171 | 6,736 | 1,086,305 | 6,924,171 / 86.44% | 267,500 / 3.34% | 818,805 / 10.22% | 285,112 / 3.56% |
| **8111** | 4,601,517 | 8,306,566 | 3,705,049 | 4,513,953 | 3,076,950 | 2,702 | 492,197 | 3,076,950 / 86.21% | 113,951 / 3.19% | 378,246 / 10.6% | 141,278 / 3.96% |
| **8112** | 4,068,506 | 8,298,865 | 4,230,359 | 4,417,368 | 2,654,239 | 1,263 | 696,027 | 2,654,239 / 79.22% | 131,542 / 3.93% | 564,485 / 16.85% | 130,998 / 3.91% |
| **8113** | 4,099,954 | 5,359,638 | 1,259,684 | 1,466,709 | 3,209,442 | 2,427 | 561,921 | 3,209,442 / 85.1% | 95,628 / 2.54% | 466,293 / 12.36% | 164,743 / 4.37% |
| **8114** | 3,823,242 | 6,971,273 | 3,148,031 | 3,298,143 | 2,686,733 | 2,344 | 598,502 | 2,686,733 / 81.78% | 97,681 / 2.97% | 500,821 / 15.24% | 166,027 / 5.05% |
| **8117** | 5,077,739 | 8,994,611 | 3,916,872 | 4,898,825 | 3,270,868 | 2,715 | 659,028 | 3,270,868 / 83.23% | 114,006 / 2.9% | 545,022 / 13.87% | 207,117 / 5.27% |
| **8118** | 6,988,036 | 9,055,869 | 2,067,833 | 2,549,855 | 5,469,202 | 7,807 | 888,282 | 5,469,202 / 86.03% | 208,657 / 3.28% | 679,625 / 10.69% | 268,349 / 4.22% |
| **8119** | 5,136,546 | 7,334,491 | 2,197,945 | 2,469,558 | 3,904,057 | 5,723 | 754,751 | 3,904,057 / 83.8% | 134,367 / 2.88% | 620,384 / 13.32% | 224,683 / 4.82% |
| **8122** | 6,790,589 | 10,563,902 | 3,773,313 | 4,794,666 | 4,858,093 | 4,048 | 690,340 | 4,858,093 / 87.56% | 164,108 / 2.96% | 526,232 / 9.48% | 181,111 / 3.26% |
| **8123** | 6,350,246 | 7,470,948 | 1,120,702 | 1,455,750 | 5,322,592 | 6,317 | 627,177 | 5,322,592 / 89.46% | 122,505 / 2.06% | 504,672 / 8.48% | 192,586 / 3.24% |
| **8124** | 6,595,776 | 8,259,208 | 1,663,432 | 2,057,532 | 5,451,836 | 6,872 | 625,939 | 5,451,836 / 89.7% | 143,668 / 2.36% | 482,271 / 7.93% | 180,290 / 2.97% |
| **8126** | 5,048,482 | 11,859,157 | 6,810,675 | 7,170,387 | 3,109,655 | 1,622 | 877,668 | 3,109,655 / 77.99% | 172,412 / 4.32% | 705,256 / 17.69% | 218,363 / 5.48% |
| **Total** | 141,365,247 | 251,265,193 | 109,899,946 | 121,813,194 | 97,590,045 | 97,095 | 20,687,054 | | | | |
| **Average** | 5,890,219 | 10,469,383 | 4,579,164 | 5,075,550 | 4,066,252 | 4,046 | 861,961 | | | | |

**Table S4.** Enriched GO terms associated with up-regulated sequences identified using Fisher's Exact Test.

| Tags | GO ID | Go Name | GO Category | P-value | No. Test set | No. Reference set | Non- Annotated Test set | Non-Annotated Reference set |
|---|---|---|---|---|---|---|---|---|
| OVER | GO:0016706 | 2-Oxoglutarate-Dependent Dioxygenase Activity | Molecular Function | 3.71E-04 | 15 | 131 | 562 | 14746 |
| OVER | GO:0004672 | Protein Kinase Activity | Molecular Function | 6.22E-04 | 71 | 1196 | 506 | 13681 |
| OVER | GO:0005524 | ATP Binding | Molecular Function | 1.92E-03 | 118 | 2300 | 459 | 12577 |
| OVER | GO:0006468 | Protein Phosphorylation | Biological Process | 0.00242 | 68 | 1196 | 509 | 13681 |
| OVER | GO:0019310 | Inositol Catabolic Process | Biological Process | 0.002519 | 3 | 5 | 574 | 14872 |
| OVER | GO:0050113 | Inositol Oxygenase Activity | Molecular Function | 2.52E-03 | 3 | 5 | 574 | 14872 |
| OVER | GO:0006915 | Apoptotic Process | Biological Process | 0.003103 | 24 | 313 | 553 | 14564 |
| OVER | GO:0004134 | 4-Alpha-Glucanotransferase Activity | Molecular Function | 0.004072 | 2 | 1 | 575 | 14876 |
| OVER | GO:0005506 | Iron Ion Binding | Molecular Function | 0.005631 | 7 | 51 | 570 | 14826 |
| OVER | GO:0043531 | ADP Binding | Molecular Function | 0.007181 | 22 | 304 | 555 | 14573 |
| UNDER | GO:0003735 | Structural Constituent of Ribosome | Molecular Function | 0.008555 | 4 | 339 | 573 | 14538 |
| UNDER | GO:0005840 | Ribosome | Cellular Component | 0.008555 | 4 | 339 | 573 | 14538 |
| UNDER | GO:0007264 | Small GTPase Mediated Signal Transduction | Biological Process | 0.023116 | 0 | 114 | 577 | 14763 |
| OVER | GO:0051087 | Chaperone Binding | Molecular Function | 0.023828 | 3 | 14 | 574 | 14863 |
| OVER | GO:0008080 | N-Acetyltransferase Activity | Molecular Function | 0.02547 | 5 | 40 | 572 | 14837 |
| OVER | GO:0005778 | Peroxisomal Membrane | Cellular Component | 0.025797 | 2 | 5 | 575 | 14872 |
| OVER | GO:0015630 | Microtubule Cytoskeleton | Cellular Component | 0.033403 | 4 | 29 | 573 | 14848 |
| OVER | GO:0007031 | Peroxisome Organization | Biological Process | 0.033556 | 2 | 6 | 575 | 14871 |
| OVER | GO:0004806 | Triglyceride Lipase Activity | Molecular Function | 0.035252 | 5 | 44 | 572 | 14833 |
| OVER | GO:0042823 | Pyridoxal Phosphate Biosynthetic Process | Biological Process | 0.037337 | 1 | 0 | 576 | 14877 |
| OVER | GO:0009097 | Isoleucine Biosynthetic Process | Biological Process | 0.037337 | 1 | 0 | 576 | 14877 |

| | | | | | | | | | |
|---|---|---|---|---|---|---|---|---|---|
| OVER | GO:0004794 | L-Threonine Ammonia-Lyase Activity | Molecular Function | 0.037337 | 1 | 0 | 576 | 14877 |
| OVER | GO:0003868 | 4-Hydroxyphenylpyruvate Dioxygenase Activity | Molecular Function | 0.037337 | 1 | 0 | 576 | 14877 |
| UNDER | GO:0006518 | Peptide Metabolic Process | Biological Process | 0.039692 | 10 | 482 | 567 | 14395 |
| OVER | GO:0016987 | Sigma Factor Activity | Molecular Function | 0.042093 | 2 | 7 | 575 | 14870 |

**Table S5.** Enriched GO terms associated with down-regulated sequences identified using Fisher's Exact Test.

| Tags | GO ID | Go Name | GO Category | P-value | No. Test set | No. Reference set | Non- Annotated Test set | Non-Annotated Reference set |
|---|---|---|---|---|---|---|---|---|
| OVER | GO:0009055 | Electron Transfer Activity | Molecular Function | 2.65E-05 | 38 | 483 | 506 | 14427 |
| UNDER | GO:0005737 | Cytoplasm | Cellular Component | 0.001862 | 9 | 623 | 535 | 14287 |
| UNDER | GO:0003677 | DNA Binding | Molecular Function | 0.002044 | 31 | 1409 | 513 | 13501 |
| UNDER | GO:0005515 | Protein Binding | Molecular Function | 0.002089 | 71 | 2697 | 473 | 12213 |
| UNDER | GO:0005524 | ATP Binding | Molecular Function | 0.002133 | 60 | 2358 | 484 | 12552 |
| UNDER | GO:0006468 | Protein Phosphorylation | Biological Process | 0.002367 | 26 | 1238 | 518 | 13672 |
| UNDER | GO:0004672 | Protein Kinase Activity | Molecular Function | 0.002384 | 26 | 1241 | 518 | 13669 |
| UNDER | GO:0005634 | Nucleus | Cellular Component | 0.002557 | 13 | 760 | 531 | 14150 |
| OVER | GO:0020037 | Heme Binding | Molecular Function | 0.003537 | 26 | 378 | 518 | 14532 |
| OVER | GO:0006570 | Tyrosine Metabolic Process | Biological Process | 0.003624 | 2 | 1 | 542 | 14909 |
| OVER | GO:0006814 | Sodium Ion Transport | Biological Process | 0.004326 | 3 | 7 | 541 | 14903 |
| OVER | GO:0006662 | Glycerol Ether Metabolic Process | Biological Process | 0.005569 | 5 | 28 | 539 | 14882 |
| OVER | GO:0016021 | Integral Component of Membrane | Cellular Component | 0.007259 | 42 | 748 | 502 | 14162 |
| OVER | GO:0055085 | Transmembrane Transport | Biological Process | 0.010387 | 37 | 648 | 507 | 14262 |
| OVER | GO:0004556 | Alpha-Amylase Activity | Molecular Function | 0.011524 | 2 | 3 | 542 | 14907 |
| OVER | GO:0016998 | Cell Wall Macromolecule Catabolic Process | Biological Process | 0.012632 | 5 | 35 | 539 | 14875 |
| OVER | GO:0015035 | Protein Disulfide Oxidoreductase Activity | Molecular Function | 0.01305 | 8 | 81 | 536 | 14829 |
| OVER | GO:0009765 | Photosynthesis, Light Harvesting | Biological Process | 0.014008 | 4 | 23 | 540 | 14887 |
| OVER | GO:0033218 | Amide Binding | Molecular Function | 0.01439 | 3 | 12 | 541 | 14898 |
| UNDER | GO:0016853 | Isomerase Activity | Molecular Function | 0.015496 | 0 | 129 | 544 | 14781 |
| OVER | GO:0015746 | Citrate Transport | Biological Process | 0.016886 | 2 | 4 | 542 | 14906 |
| OVER | GO:0015137 | Citrate Transmembrane Transporter Activity | Molecular Function | 0.016886 | 2 | 4 | 542 | 14906 |
| OVER | GO:0004197 | Cysteine-Type Endopeptidase Activity | Molecular Function | 0.020416 | 3 | 14 | 541 | 14896 |
| OVER | GO:0016117 | Carotenoid Biosynthetic Process | Biological Process | 0.023094 | 2 | 5 | 542 | 14905 |
| OVER | GO:0006821 | Chloride Transport | Biological Process | 0.023094 | 2 | 5 | 542 | 14905 |
| OVER | GO:0005247 | Voltage-Gated Chloride Channel Activity | Molecular Function | 0.023094 | 2 | 5 | 542 | 14905 |
| OVER | GO:0005452 | Inorganic Anion Exchanger Activity | Molecular Function | 0.023094 | 2 | 5 | 542 | 14905 |
| OVER | GO:0016844 | Strictosidine Synthase Activity | Molecular Function | 0.030082 | 2 | 6 | 542 | 14904 |
| OVER | GO:0005975 | Carbohydrate Metabolic Process | Biological Process | 0.033813 | 35 | 657 | 509 | 14253 |
| OVER | GO:0006559 | L-Phenylalanine Catabolic Process | Biological Process | 0.035201 | 1 | 0 | 543 | 14910 |
| OVER | GO:0009252 | Peptidoglycan Biosynthetic Process | Biological Process | 0.035201 | 1 | 0 | 543 | 14910 |
| OVER | GO:0006817 | Phosphate Ion Transport | Biological Process | 0.035201 | 1 | 0 | 543 | 14910 |
| OVER | GO:0006379 | mRNA Cleavage | Biological Process | 0.035201 | 1 | 0 | 543 | 14910 |
| OVER | GO:0030328 | Prenylcysteine Catabolic Process | Biological Process | 0.035201 | 1 | 0 | 543 | 14910 |
| OVER | GO:0000213 | tRNA-Intron Endonuclease Activity | Molecular Function | 0.035201 | 1 | 0 | 543 | 14910 |
| OVER | GO:0015385 | Sodium:Proton Antiporter Activity | Molecular Function | 0.035201 | 1 | 0 | 543 | 14910 |
| OVER | GO:0004411 | Homogentisate 1,2-Dioxygenase Activity | Molecular Function | 0.035201 | 1 | 0 | 543 | 14910 |
| OVER | GO:0005315 | Inorganic Phosphate Transmembrane Transporter Activity | Molecular Function | 0.035201 | 1 | 0 | 543 | 14910 |
| OVER | GO:0003864 | 3-Methyl-2-Oxobutanoate Hydroxymethyltransferase Activity | Molecular Function | 0.035201 | 1 | 0 | 543 | 14910 |
| OVER | GO:0000172 | Ribonuclease MRP Complex | Cellular Component | 0.035201 | 1 | 0 | 543 | 14910 |
| OVER | GO:0030677 | Ribonuclease P Complex | Cellular Component | 0.035201 | 1 | 0 | 543 | 14910 |
| UNDER | GO:0004386 | Helicase Activity | Molecular Function | 0.036707 | 2 | 209 | 542 | 14701 |

| | | | | | | | | |
|---|---|---|---|---|---|---|---|---|
| OVER | GO:0045454 | Cell Redox Homeostasis | Biological Process | 0.041671 | 9 | 114 | 535 | 14796 |
| OVER | GO:0016717 | Oxidoreductase Activity, Acting on paired Ddnors, with oxidation of a pair of donors resulting in the reduction of molecular oxygen to two molecules of water | Molecular Function | 0.046154 | 2 | 8 | 542 | 14902 |
| OVER | GO:0004869 | Cysteine-Type Endopeptidase Inhibitor Activity | Molecular Function | 0.046154 | 2 | 8 | 542 | 14902 |
| OVER | GO:0008889 | Glycerophosphodiester Phosphodiesterase Activity | Molecular Function | 0.046154 | 2 | 8 | 542 | 14902 |
| OVER | GO:0006857 | Oligopeptide Transport | Biological Process | 0.048397 | 6 | 69 | 538 | 14841 |

**Table S6.** Gene list of all the top 50 differentially expressed genes (DEGs) with a distinct expression pattern in the slow-cooking (Pinto) and fast-cooking (Rosecoco) bean varieties.

| No | Gene ID | Log2 fold change | P-value |
|---|---|---|---|
| 1 | *Phvul.001G068400* | -4.7152 | 2.91E-18 |
| 2 | *Phvul.001G114880* | -4.0605 | 2.12E-14 |
| 3 | *Phvul.002G273200* | 3.18511 | 3.01E-14 |
| 4 | *Phvul.003G047300* | 6.64992 | 2.54E-15 |
| 5 | *Phvul.003G053900* | -2.5894 | 3.40E-13 |
| 6 | *Phvul.003G054300* | 4.89515 | 1.24E-15 |
| 7 | *Phvul.003G091800* | -3.9939 | 6.23E-15 |
| 8 | *Phvul.003G213500* | 2.99167 | 3.26E-15 |
| 9 | *Phvul.003G257900* | -2.5961 | 4.40E-18 |
| 10 | *Phvul.003G285800* | 5.49203 | 2.91E-17 |
| 11 | *Phvul.004G012900* | -3.8354 | 3.03E-13 |
| 12 | *Phvul.004G085694* | 4.86012 | 5.42E-15 |
| 13 | *Phvul.004G158200* | -7.748 | 2.22E-14 |
| 14 | *Phvul.005G062000* | 8.86455 | 2.29E-40 |
| 15 | *Phvul.005G063900* | 8.36892 | 4.66E-35 |
| 16 | *Phvul.005G086200* | 4.97535 | 5.92E-14 |
| 17 | *Phvul.006G029700* | -4.7696 | 6.25E-15 |
| 18 | *Phvul.006G054466* | -6.6075 | 5.00E-38 |
| 19 | *Phvul.006G088600* | -3.5753 | 2.84E-14 |
| 20 | *Phvul.006G133200* | 3.71225 | 1.20E-15 |
| 21 | *Phvul.006G174000* | -3.942 | 3.78E-17 |
| 22 | *Phvul.007G033301* | -5.9174 | 2.62E-26 |
| 23 | *Phvul.007G040700* | -4.0795 | 2.83E-15 |
| 24 | *Phvul.007G070200* | -7.5921 | 1.31E-13 |
| 25 | *Phvul.007G141850* | 3.39405 | 4.86E-19 |
| 26 | *Phvul.007G163950* | -5.8063 | 2.27E-16 |
| 27 | *Phvul.007G170900* | -6.4766 | 9.83E-20 |
| 28 | *Phvul.007G218500* | -4.3622 | 1.74E-15 |
| 29 | *Phvul.007G246100* | -4.8454 | 1.02E-16 |
| 30 | *Phvul.007G278000* | 4.78928 | 7.00E-16 |
| 31 | *Phvul.008G027600* | 3.37788 | 8.85E-15 |
| 32 | *Phvul.008G109466* | -6.4092 | 1.10E-19 |
| 33 | *Phvul.008G121300* | -4.5949 | 3.52E-21 |
| 34 | *Phvul.008G127537* | -5.3512 | 4.20E-30 |
| 35 | *Phvul.008G133200* | 9.62583 | 2.02E-20 |
| 36 | *Phvul.008G141500* | 3.56155 | 6.01E-21 |
| 37 | *Phvul.008G209214* | -6.8952 | 3.11E-33 |
| 38 | *Phvul.008G244800* | -5.3258 | 1.42E-13 |
| 39 | *Phvul.008G265300* | -5.5312 | 1.74E-16 |
| 40 | *Phvul.008G268500* | 4.70487 | 1.80E-14 |

| | | | |
|---|---|---|---|
| 41 | *Phvul.009G049100* | -4.3287 | 2.28E-14 |
| 42 | *Phvul.009G134300* | -3.7001 | 1.37E-18 |
| 43 | *Phvul.009G158600* | -5.9323 | 1.73E-14 |
| 44 | *Phvul.009G191800* | 3.82083 | 2.48E-18 |
| 45 | *Phvul.009G260300* | -3.0615 | 7.88E-18 |
| 46 | *Phvul.010G019350* | 7.11799 | 1.97E-13 |
| 47 | *Phvul.010G050300* | -7.2856 | 2.53E-44 |
| 48 | *Phvul.010G062700* | -6.8449 | 4.50E-20 |
| 49 | *Phvul.010G121301* | -4.6291 | 8.43E-16 |
| 50 | *Phvul.011G175950* | -5.4382 | 1.94E-14 |

Gene identifiers (ID) are obtained from the PhytoMine tool available on the Phytozome database [42]. Genes numbered from 38 to 50 have no known functional annotation in the database. Log-CPM represents the logarithmic value of normalized reads in counts per million (CPM).

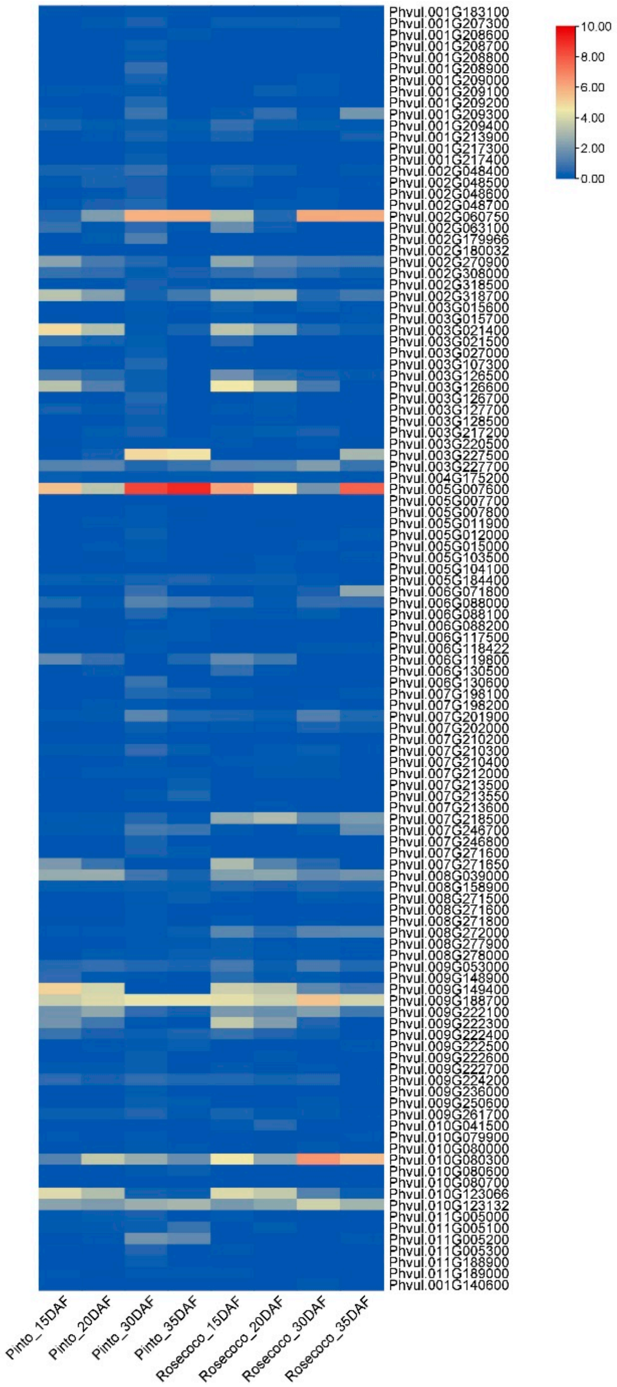

**Figure S1.** Heat map showing the expression of genes encoding pectin methyl-esterases (PMEs) and pectin-methyl-esterase inhibitors (PMEIs) in a slow-cooking (Pinto) and a fast-cooking (Rosecoco) *P. vulgaris* variety. Normalized scaling was applied to rows and the intensity of color associated with gene expression ranges from blue (low expression) to red (high expression). The gene identifiers (IDs) are shown on the right side of the figure while the bean varieties and sampled development stages (Days after Flowering- DAF) are shown at the bottom. Heat map was drawn using TBtools software [36].

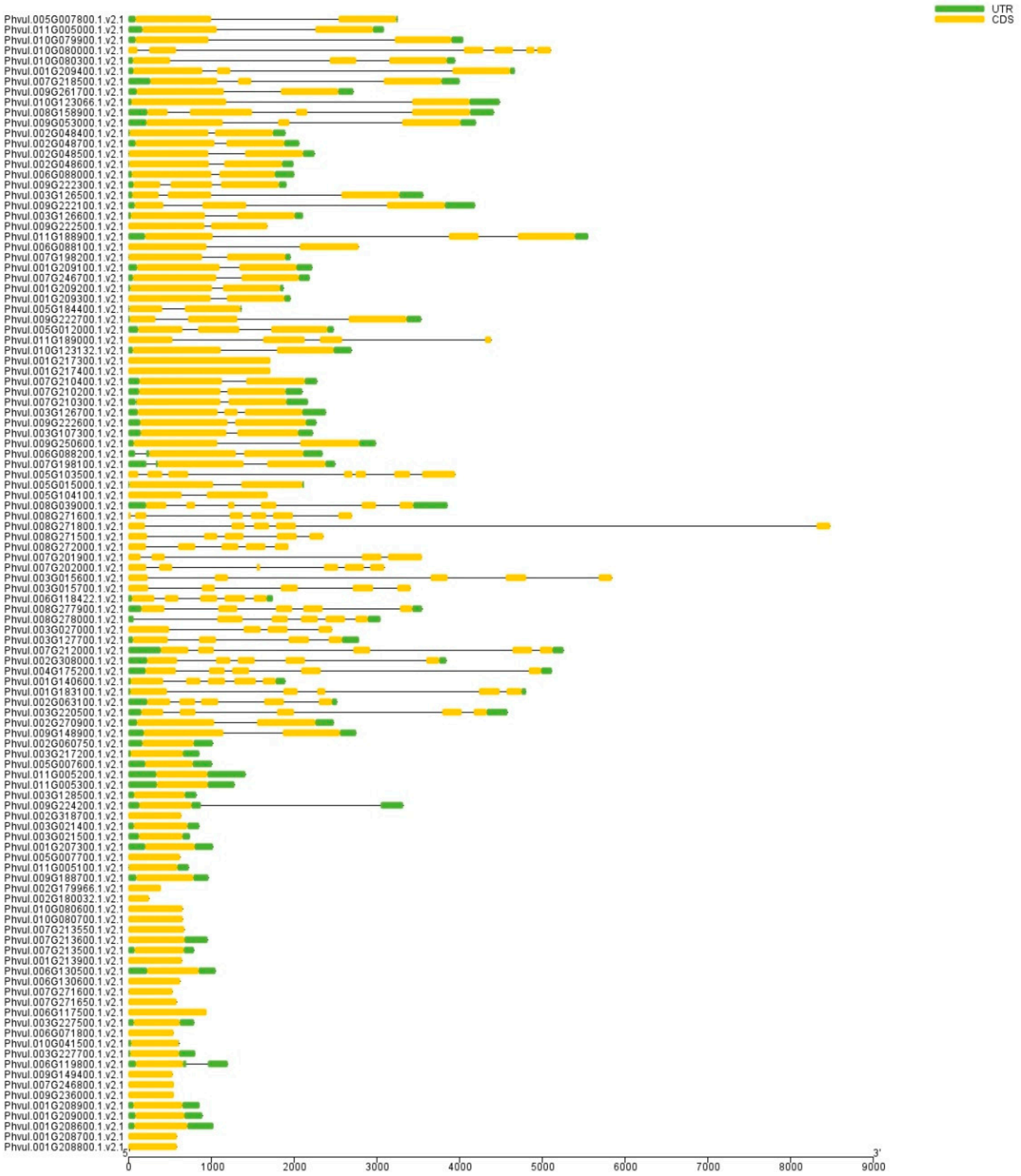


**Figure S2.** Exon-intron structure of genes encoding the pectin methyl-esterase and pectin methyl-esterase inhibitors in *P. vulgaris*. Gene structures were visualized using GSDS software [92].
